# Probing the Functional Impact of Sequence Variation on p53-DNA Interactions Using a Novel Microsphere Assay for Protein-DNA Binding with Human Cell Extracts

**DOI:** 10.1371/journal.pgen.1000462

**Published:** 2009-05-08

**Authors:** Maher A. Noureddine, Daniel Menendez, Michelle R. Campbell, Omari J. Bandele, Monica M. Horvath, Xuting Wang, Gary S. Pittman, Brian N. Chorley, Michael A. Resnick, Douglas A. Bell

**Affiliations:** 1 Environmental Genomics Group, Laboratory of Molecular Genetics, National Institute of Environmental Health Sciences, Research Triangle Park, North Carolina, United States of America; 2 Chromosome Stability Group, Laboratory of Molecular Genetics, National Institute of Environmental Health Sciences, Research Triangle Park, North Carolina, United States of America; Stanford University School of Medicine, United States of America

## Abstract

The p53 tumor suppressor regulates its target genes through sequence-specific binding to DNA response elements (REs). Although numerous p53 REs are established, the thousands more identified by bioinformatics are not easily subjected to comparative functional evaluation. To examine the relationship between RE sequence variation—including polymorphisms—and p53 binding, we have developed a multiplex format microsphere assay of protein-DNA binding (MAPD) for p53 in nuclear extracts. Using MAPD we measured sequence-specific p53 binding of doxorubicin-activated or transiently expressed p53 to REs from established p53 target genes and p53 consensus REs. To assess the sensitivity and scalability of the assay, we tested 16 variants of the p21 target sequence and a 62-multiplex set of single nucleotide (nt) variants of the p53 consensus sequence and found many changes in p53 binding that are not captured by current computational binding models. A group of eight single nucleotide polymorphisms (SNPs) was examined and binding profiles closely matched transactivation capability tested in luciferase constructs. The in vitro binding characteristics of p53 in nuclear extracts recapitulated the cellular in vivo transactivation capabilities for eight well-established human REs measured by luciferase assay. Using a set of 26 bona fide REs, we observed distinct binding patterns characteristic of transiently expressed wild type and mutant p53s. This microsphere assay system utilizes biologically meaningful cell extracts in a multiplexed, quantitative, in vitro format that provides a powerful experimental tool for elucidating the functional impact of sequence polymorphism and protein variation on protein/DNA binding in transcriptional networks.

## Author Summary

Characterizing the functional roles of gene regulatory sequences and the impact of genetic polymorphism on protein-DNA binding are rapidly growing areas of genomics. The p53 tumor suppressor regulates its target genes through sequence-specific binding to DNA response elements, and we have developed a multiplex format microsphere assay (MAPD) to probe how sequence variation alters p53 binding. Testing pools of multiplexed oligonucleotides that contain numerous p53 binding targets or different sequence variants, we demonstrate that subtle changes in target sequence can impact p53 binding, and these effects are not captured by commonly used computational binding models. A group of single nucleotide polymorphisms was examined and binding profiles closely matched gene expression measured in a parallel system. We also observed distinct binding patterns that were characteristic of transiently expressed wild type and mutant p53s, suggesting that this method may be useful for probing the impact of protein structural variation on DNA binding. MAPD utilizes biologically meaningful cell extracts in a multiplexed, quantitative format that provides a powerful experimental tool for elucidating the functional impact of sequence polymorphism and protein variation on protein/DNA binding in transcriptional networks.

## Introduction

The prominent tumor suppressor p53 is a transcription factor (TF) that regulates a vast network of genes. Functional evaluation of binding to its target DNA response elements (REs) is a key step in understanding the many events that can lead to tumorigenesis. Following activation, p53 localizes to the nucleus, forms a tetramer, binds to REs and transactivates or suppresses target genes[1],[2]. The well-established p53 RE consensus sequence consists of two 10-nt half-sites (RRRCWWGYYY-N*-RRRCWWGYYY, R = purine, Y = pyrimidine, W = A/T and N* = 0 to 13-nt spacer) [3] and p53 binding to these DNA elements is a necessary event for p53-regulated gene expression. The relationship between p53 activation, binding and in vivo gene transactivation remains an important area of investigation [4]. Experimental studies have identified numerous bona fide REs necessary for p53-regulated transactivation, and thousands of potential sites have been implicated by bioinformatics [5],[6]. The presence of single nucleotide polymorphisms (SNPs) in REs may impact transcription factor binding and result in altered gene expression. Genetic variation in TF binding is a significant evolutionary mechanism in mammals [7] and directs species-specific gene expression [8]. In human populations, response element variation is known to alter cellular processes including diabetes [9], development [10], angiogenesis [11] and susceptibility to many other diseases including cancer [12]. With the accelerating identification of noncoding, disease- and expression-associated SNPs through genome-wide association studies [13], evaluating the functional and mechanistic consequences of regulatory SNPs is a general problem in human genetics. We have identified several hundred SNPs in the human genome that reside within putative p53 REs [5]. Assessing the impact of these RE SNPs is of direct relevance to p53 function in the cell cycle, DNA repair, apoptosis and subsequent tumorigenesis.

Binding of p53 or its mutants to REs has been assessed by in vitro techniques such as electrophoretic mobility shift (EMSA), surface plasmon resonance, fluorescent anisotropy and chromatin immunoprecipitation (ChIP) but each of these approaches has experimental limitations. We sought to test p53 binding to a large number of defined sequences, including single nucleotide variants, with cell extracts generated from different experimental conditions using an approach that would be amenable to high sample throughput and replication. Microsphere-based flow cytometric assays utilize fluorescent-dyed beads (∼6 micrometer microspheres) coated with molecules such as nucleic acids or peptides [14]. Beads are placed in an assay medium containing biological targets (e.g., cell extracts containing p53 or ERα) where interactions can take place. Each bead type has a distinct spectral signature and up to 100 bead types per reaction can be used. A dual laser-equipped flow cytometer detects both the bead identity and a fluorescent reporter dye attached to the target of interest that is captured on the bead. The fluorescence signal intensity reflects relative quantification of target/bead interaction. This technology has been adapted to many assays [15], including RNA expression analysis [16], serum cytokine analysis [17],[18] and high-throughput genotyping [18],[19],[20],[21].

Based on this platform, we developed a highly reproducible general approach for performing large-scale quantitative studies of protein-DNA binding using p53 or other transcription factors, such as ERα Importantly, this microsphere assay for protein-DNA binding (MAPD) generates biologically-meaningful binding data using nuclear extracts from human cells, permitting flexible experimental designs. We show that the MAPD approach provides high resolution and utility in the functional evaluation of p53 binding to RE variants, including single nucleotide changes in REs, making it an effective tool in modern genomics research. Comparing experimental and computed binding values based on a position weight matrix (PWM) model, commonly used for evaluating TF binding, we demonstrate that subtle changes in the binding sequence have functional consequences that are not captured by PWM models. Furthermore, using a panel of p53 REs and p53 RE SNPs, we observed patterns of binding that are highly correlated with luciferase-based transactivation measurements in mammalian cells. The p53 mutants, S121F and R175H, displayed a change in binding specificity and a loss of binding respectively. The binding properties of these mutants are consistent with their previously described effects on gene expression [22]. While in vitro binding measurements are not a surrogate for ChIP or gene expression studies, this method allows quantification of a necessary step in transcription. This work demonstrates that MAPD can not only be used to evaluate RE sequence variation and SNPs, but may also be useful for probing functional changes in the protein structure of DNA binding proteins.

## Results

### Multiplex Binding Measurements of Activated p53 with Known REs

**Figure 1:**
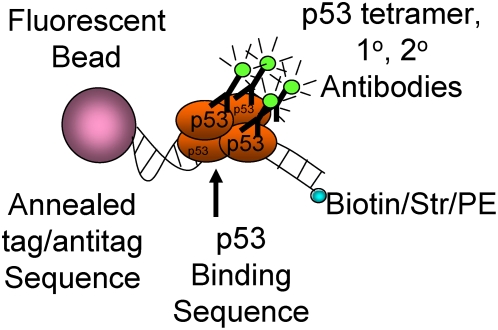
Microsphere assay for protein-DNA (MAPD) binding. Double-stranded oligonucleotides containing p53 binding sequence of interest and single-stranded tag overhang were hybridized to FlexMap beads bearing complementary anti-tag sequence. Multiplex groups of beads were prepared and incubated with nuclear extracts containing transcription factor. Primary and secondary antibodies were added, beads were sorted and fluorescence signal was quantified for each bead type and normalized by biotin/streptavidin/phycoerythrin signal to adjust for bead-specific signal effects.

The effects of sequence variation among binding sites for a given TF have been difficult to quantify in protein-DNA binding experiments. We developed a fluorescent microsphere-based approach to interrogate the interaction between p53 and its target REs (Figure 1 and S1). Double-stranded DNA fragments bearing p53 RE sequences (ConA, ConC, p21, PUMA, GADD45) and a negative control (WRNC) were uniquely linked to different sets of fluorescent microsphere beads (see Materials and Methods and Supporting Information for oligonucleotide design). Equal quantities of the 6 bead types were combined. The p21 RE, a strong binding sequence, was used as a positive control. ConA and ConC are both perfect matches with the p53 consensus RE and have been demonstrated to function as strong binding REs in a p53 model system [23] and human cells (Menendez, Jordan and Resnick, unpublished) with ConA being more responsive to p53. p53 binding to the strong RE from p21 and to the moderate-to-weak REs from PUMA and GADD45, respectively, are well established [24],[25].

**Figure 2:**
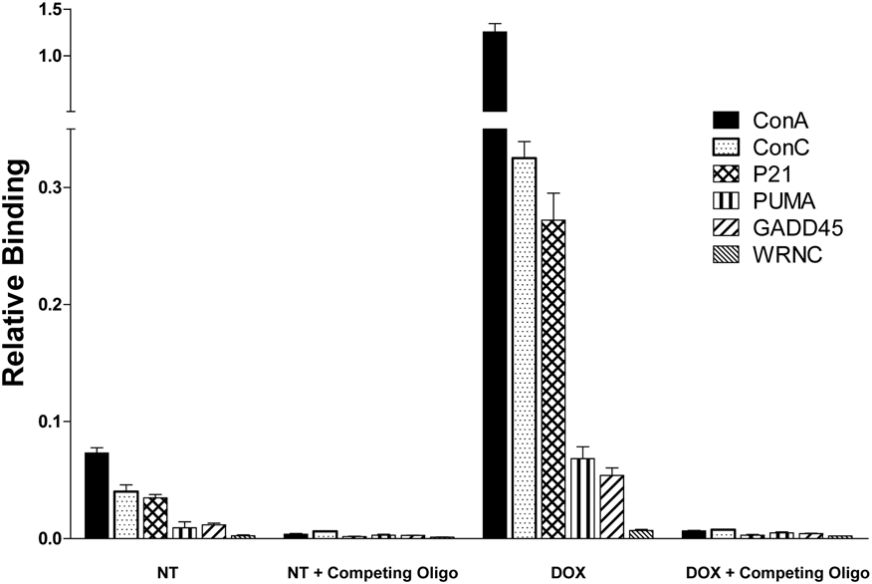
Detection of binding interactions between activated p53 and a set of multiplexed REs. A six-plex set of oligonucleotide-conjugated beads, carrying p53 binding sites (ConA, ConC, p21, PUMA, GADD45) and a negative control (WRNC) were incubated with nuclear extracts for 60 minutes, and p53 binding was measured. Nuclear extracts were from non-treated lymphoblast cells (NT) or cells treated with 1 mM doxorubicin (DOXO) for 24 hrs. Addition of a competing oligonucleotide demonstrates binding specificity. Relative binding was obtained for each RE tested. The split vertical scale allows display of data from high and low values. Values shown are the means for each bead type+/−SD (n = 3).

Figure 2 displays p53 binding after incubating the 6-plex beads with nuclear extracts from human lymphoblastoid cells that were either not treated (NT), containing small amounts of activated p53, or treated with doxorubicin (DOXO), inducing high levels of activated p53 [26]. MAPD could detect p53 binding in both. The data are corrected for bead-specific signal effects by measurement of a biotinylated reverse oligonucleotide on the same bead/oligo construct conjugated with phycoerythrin/streptavidin as described in the Materials and Methods. Binding values are relative to the biotin signal. The ConA sequence (black bar) showed appreciable binding (0.073+/−0.0045) in the NT extract, and this level was ∼30-fold greater than the negative control (WRNC: 0.0023+/−0.0009). Relative to the NT values, nuclear extracts from DOXO-treated cells had a 17-fold increase for ConA (1.26+/−0.089), an 8-fold increase for p21 (0.27+/−0.023) and a 5-fold increase for weak-binding GADD45 (0.054+/−0.006). The DOXO-induced ConA signal was ∼180-fold greater than the DOXO negative control value. For GADD45 there was an 8-fold greater signal over the negative control, demonstrating the large dynamic range of the assay for both strong and weak binding sequences.

To examine binding specificity, the bead set was presented to nuclear extracts (NT and DOXO) along with excess of oligonucleotide designed to directly compete for binding (Figure 2, Competing Oligo). Specificity for p53 binding was clearly demonstrated by abolishment of the signal in extracts from both NT and DOXO (see also Figures S2 and S3). An excess amount of a noncompeting oligonucleotide was used to maximize signal by blocking nonspecific binding (see optimization in Figures S2, S3). MAPD detected DOXO-activated p53 binding over a broad dynamic range (∼2.5 logs) and nuclear extract concentrations (Figure S4). Using extracts with different quantities of p53 allows quantitative comparisons of biochemical properties (Table within Figure S4). To examine the general applicability of MAPD for measuring protein-DNA binding, we also created beads bearing different estrogen receptor α binding elements and measured ERα binding. ERα binding data is presented in the Supporting Information (Figure S5A,B).

### Impact of Sequence Variation on p53 Binding

**Figure 3:**
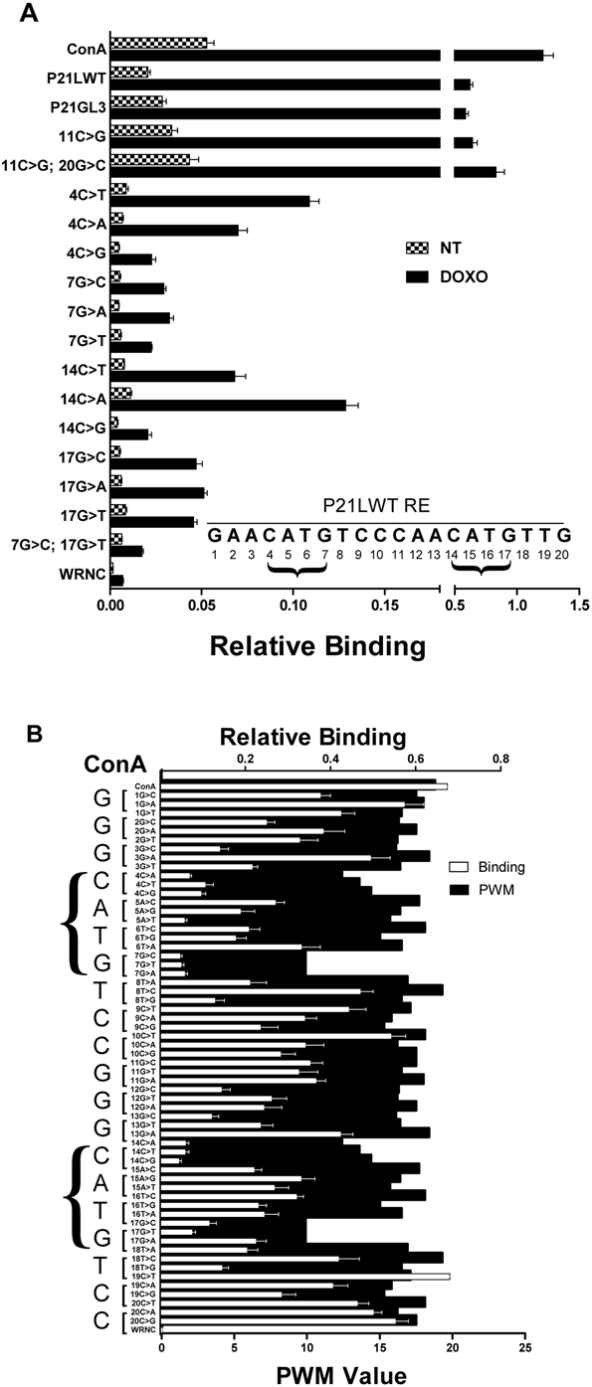
Sequence variation affects p53 binding. A) Evaluating the impact of single nucleotide variations in p21 RE on p53 binding using nuclear extracts from untreated and DOXO-treated cells. A set of 19 oligonucleotides, each conjugated to a unique bead, were multiplexed and treated with 1.75 µg of non-treated nuclear extracts (NT: checkered bar) or activated nuclear extracts (black bar: DOXO-treated lymphoblastoid cells). Relative binding intensity is shown on the horizontal axis (note the break in the scale to accommodate the signal from all oligonucleotides). Relative binding values were obtained for each oligonucleotide as discussed in Materials and Methods. The oligonucleotide bearing the p53 consensus sequence (ConA) is first on the list (top left Y-axis) followed by wild type p21RE (p21LWT). p21GL3 bears the same 20-nt RE as p21LWT but with a synthetic backbone sequence from pGL3 vector. Fifteen single or double variants of p21 RE are shown. The p21 sequence is shown as inset. The number denotes the numerical position of the base within the p21 RE. Arrowheads point to the nts that have been chosen for alteration. WRNC is a negative control oligonucleotide and carries a nonbinding sequence in the same backbone as the other oligonucleotides shown with the exception of P21GL3. Values shown are the mean+/−SD (n = 3). B) Multiplex binding measurements of 60 variants of a p53 consensus sequence. Sixty oligonucleotides, each bearing a single nucleotide variation of the p53 consensus binding site (ConA = GGGCATGTCCGGGCATGTCC) were generated. ConA sequence is shown in large letters under arrow. CATG core of each half-site is shown in a right-facing bracket. Systematic base substitutions at all positions within ConA are shown in small letters. For instance, 1G>C is an RE with the first G converted to a C. In addition, a positive control bead (ConA RE) and a negative control bead (WRNC: lacks a p53 binding RE) were included. Equal amounts of each microsphere type were mixed to generate a multiplex of 62 bead types. Following treatment with activated nuclear extracts (DOXO-treated or untreated lymphoblastoid cells, Figure S3), the relative binding intensity (value shown on upper horizontal axis) was obtained for each oligonucleotide (white bar) as discussed in Materials and Methods. Bar values are means for each bead type+/−SD (n = 3). The black bar graph in the background (*e.g.* black bars) shows the calculated PWM value for each variant oligonucleotide tested (scale depicted on the lower horizontal axis). A list of PWM values for REs used can be found in Supporting Information.

MAPD is highly suited to experiments such as site-directed mutagenesis of REs or SNP functional analysis, as shown in Figure 3A for p53 RE of p21, designated p21LWT. To investigate the importance of individual nucleotides on p53 binding, NT and DOXO-treated cell nuclear extracts were hybridized with 16 variants of the p21 RE in a 19-plex reaction that included two positive controls (ConA and p21LWT) and a negative control (WRNC). DOXO-treated cell extracts produced higher binding for all sequences. The p21 RE produced strong binding signals relative to WRNC, both when embedded in its native flanking sequence (p21LWT 0.62+/−0.021) or an unrelated flanking sequence (p21GL3 0.58+/−0.021; sequences are listed in Supporting Information). The perfect consensus p53 RE, ConA, showed a stronger signal (1.21+/−0.083) relative to p21LWT. Changing the p21LWT sequence at nt-11 from C to G (Figure 3A 11C>G), a change favoring consensus, resulted in binding that was slightly higher (0.64+/−0.038) than the native p21LWT. Moreover, when positions nt-11 and nt-20 in p21LWT were simultaneously changed to better match the consensus (Figure 3A, 11C>G; 20G>C), there was a 29% increase in p53 binding (from 0.64 to 0.83+/−0.069). This provides an example of how sequence variation has the potential to increase transcription factor binding. Among mammalian p53 REs, the most conserved positions are C and G nucleotides 4, 7, 14, and 17, and many studies have observed that changes at these positions strongly impact p53 transactivation [3],[4],[5],[6],[27],[28],[29]. The present data demonstrate that these effects are due to decreased p53 binding.

### Experimental Support for Computational Binding Models

TFBS predictions using position weight matrix (PWM) calculations are ubiquitous in the genomics literature. Binding sequences gleaned from publications (or a database such as TRANSFAC) are used to create a PWM to evaluate and score new sequences. Such statistical models determine the nucleotide frequency in a sequence motif database under the simplifying assumption that the sampled sequences are bona fide functional REs with similar binding properties and all nts act independently. Experimental quantification of TF binding strength has been laborious. Importantly, while TF binding strength is expected to greatly influence function of the p53-regulated network, or other networks, this information is rarely incorporated into binding models or RE prediction. To demonstrate the impact of SNPs on p53 binding and to show how quantitative binding data may be useful for refining RE prediction models, we generated a set of 60 oligonucleotides, each bearing a single nt variant of ConA along with positive and negative controls to yield a 62-plex experiment.

**Figure 4:**
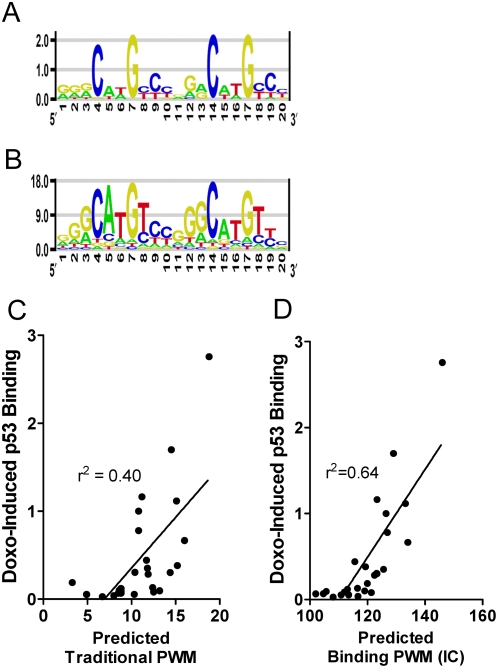
Sequence logos for p53 consensus created from two datasets. The height of the letter indicates the frequency or binding preference at that nucleotide position in the RE. A) Traditional position weight matrix with p53 RE sequences from Table S2. B) Sequence logo developed from the binding data (Figure 3B) using a modified approach of Veprintsev and Fersht [30]. Binding values were put into a binding position weight matrix (BPWM) and the letter height represents experimentally determined binding preference. C) Binding values for bona fide p53 RE sequences (as in Figure 6 and Table S1) plotted against traditional PWM model prediction (4C) and binding based model prediction (4D). The correlation between PWM calculation and measured binding improved from r^2^ = 0.40 with the traditional PWM model (Figure 4C) to r^2^ = 0.64 with binding-based model (4D).

Figure 3B displays p53 binding to the 62 oligonucleotides (Figure S6 shows NT binding) and clearly reveals differences in the impact of both the position and type of base-change (*e.g.*, purine to purine, purine to pyrimidine) and also differences from the PWM model prediction. Comparing PWM data (black bars) to experimental data (open bars) for the core motif (4C, 7G, 14C, 17G, brackets), there is a general similarity in pattern, but for many base-changes, the correlation of PWM with binding is poor. For example, in the PWM model, changes at positions 3G>C, 8T>G, 12G>C, or 18T>G have little effect (<20%), but our results show a >75% binding reduction at these positions. It is clear that PWM values, which vary by only 2-fold cannot provide a robust prediction of binding, which varies as much as 13-fold just among these sequences. Since sequences used as input to PWM models typically have been evaluated in qualitative binding assays such as EMSA, it is not surprising that PWM models often have limited predictive ability. Using the technique of Veprintsev and Fersht [30] we have generated a sequence logo from the binding data that can be compared with the traditional consensus sequence (Figure 4). The sequence logo derived from binding data differs from the traditional PWM model consensus, yet the pattern for the natively DOXO-activated p53 in nuclear extracts is generally consistent with the recent in vitro half-site binding analysis using a purified, ultra-stable mutant p53 and fluorescent anisotropy [30]. The binding-based PWM supports a strong role for C and G nucleotides at positions 4, 7, 14, 17 and also for G at position 3. The A at position 5 is characteristic of very strong p53 binding REs. In addition, these data were generated using 20mers and appear to support binding strength asymmetry within the half-site (nt 1–5 versus nt 6–10) and also asymmetry across the two half-sites, as suggested by the p53 ChIP data of Wei et al [31] and the model of Riley et al [28]. Incorporating detailed experimental binding data will allow for more accurate computational binding models. Preliminary evidence for improved model building is demonstrated in Figure 4. The binding-based PWM model was tested on the panel of bona fide RE sequences listed in Table S1. Experimental binding values for these REs are plotted versus predicted binding values using the traditional PWM (4C) and binding-based PWM (4D) models. The correlation coefficient is considerably improved (from r^2^ = 0.40 to r^2^ = 0.64) by the use of binding data in the position weight matrix model. While this represents a limited experimental data set and a simple linear model, it suggests that binding information may be useful for informing the identification of binding sites. We suggest that experiments examining a large set of strong to weak p53 REs (with various quarter-site orientations, insertions/deletions and spacer lengths) will provide data that can be incorporated into more sophisticated computational models, such as those proposed by Veprintsev and Fersht [30], and Riley, et al [28], and this should greatly improve p53 binding site prediction.

### Evaluation of SNPs in p53 Response Elements

**Figure 5:**
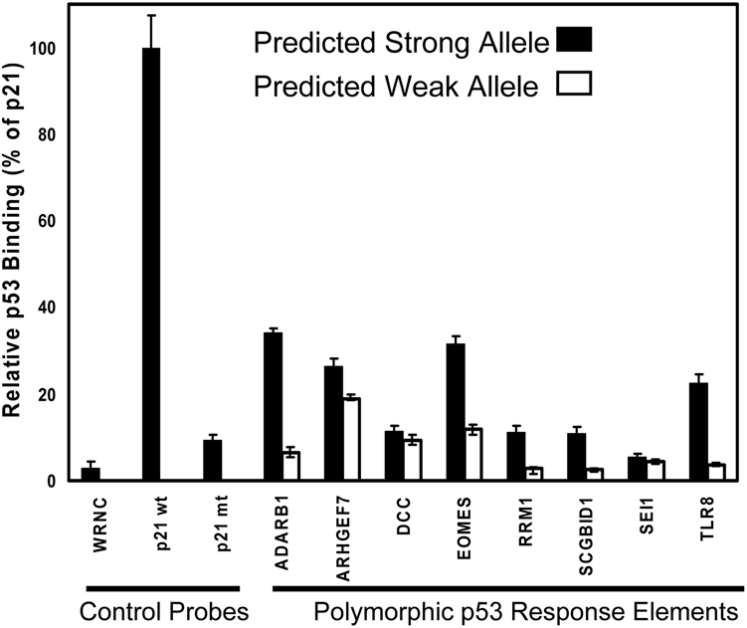
Evaluation of p53 binding to response element SNP alleles. Binding for predicted strong (Dark bars) and weak (open bars) alleles of the polymorphic sequence is shown relative to p21 RE binding (100%). Each bar represents the mean of 3 experiments carried out on different days and performed in triplicate (error bars represent SD). Positive and negative control oligonucleotides (left side) were included in each experiment and each sample was tested with a negative control extract (data not shown). This experiment was replicated using p53 extracts generated from a second tissue type (U2OS cells, Figure S8). These results are in close agreement with evaluation of these SNP alleles when cloned into a luciferase construct and transfected into SaOS2 cells expressing p53 (Tomso et al. [5]).

**Figure 6:**
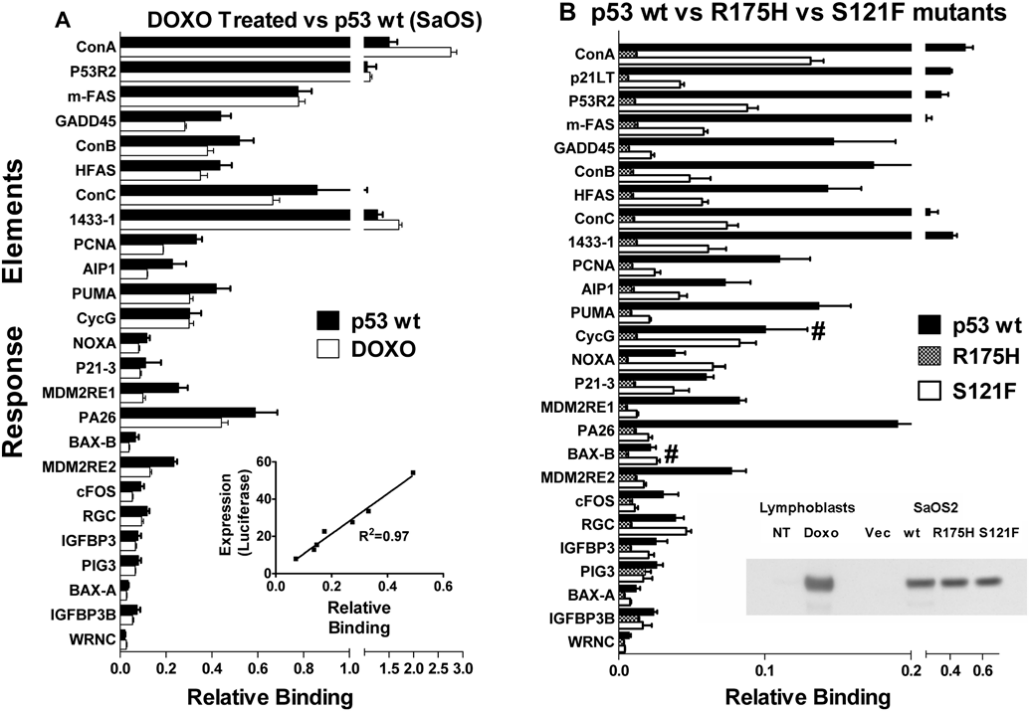
Functional interrogation of bona fide p53 REs. A multiplex oligonucleotide panel of previously validated p53 REs [23] was assembled (each oligonucleotide type on a unique microsphere bead) and tested for p53 binding. A) RE panel was treated with nuclear extracts from DOXO-treated lymphoblastoid cells or SaOS-2 cells (p53-null) that were transiently transfected with plasmid pC53-SN3 (coding for human wt p53 cDNA under control of the CMV promoter; see Materials and Methods). Equivalent quantities of p53 were used in each assay (as measured by Western (shown in insert panel 6B) and ELISA). Following normalization to p21LWT value (p21 = 1), patterns of binding are similar for DOXO activated p53 and ectopically expressed wt p53 (see also Figure S7A). The insert in Figure 6A displays the correlation between binding and luciferase expression for several elements. Figure S7 displays the relationship between DOXO activated p53 binding and p53 ChIP. The panel included ConA and WRNC as positive and negative controls, respectively. B) RE panel of beads was treated with nuclear extracts from either SaOS-2 cells (p53-null) that have been transiently transfected with empty plasmid (p53 null, not shown), plasmid expressing p53 wt (p53 wt), plasmids expressing p53 mutant S121F or R175H. Equivalent quantities of p53 were used in each assay (as measured by Western (shown in insert panel 6B); no binding was observed for p53−/− SaOS-2 cells. Symbol (#) indicates RE retained binding in the S121F mutant. Bar values are mean±SEM (n = 3). Note both the numerical difference and the breaks in the scale of horizontal axes.

SNPs in 8 putative p53 REs were identified using the bioinformatics approach described in Tomso et al [5]. Sequences are shown in Table S1. Oligonucleotides containing the predicted weak and strong alleles for these p53 REs were placed on microspheres and evaluated for binding to activated p53 in nuclear extracts (Figure 5). Negative and positive control oligonucleotides were hybridized in parallel in the multiplexed reaction and nuclear extract from untreated cells was also tested as a negative control (mean binding to p21 probe for untreated cell extracts was 1.6%+/−0.19%, n = 6 of the Doxo treated value); binding to p21 was consistent with p53 content assessed by western blot and TransAm ELISA. Among these putative p53 REs, the predicted strong alleles for REs in ADARB1, ARHGEF7, and EOMES displayed binding strength that was ∼30% of p21 binding, a level that is similar to PUMA and GADD45 (Figures 2 and 6). With the exception of REs upstream of DCC and SEI1, we observed that the predicted weak alleles showed significantly reduced binding. SNPs that altered the conserved core sequence (ADARB1, EOMES, RRM1 and TLR8) showed dramatic reductions in binding. This approach to functional evaluation of TF binding site SNPs would be useful for rapid screening of candidate SNPs in TF binding sites identified through bioinformatics or whole genome association studies.

### Assessing the Binding Spectrum Phenotype of Wild Type vs. Mutant p53 Proteins

The p53 protein is a master regulator of the cell cycle, DNA repair and apoptosis. p53 activation state is dependent on tissue type and activating stimulus [2] and may impact binding, but few experiments have explored this idea. Using a panel of p53 target REs to assess binding phenotype, we examined nuclear extracts containing activated p53 from different human cell types (Figure 6A). To compare the p53 binding spectra for these functional p53 molecules, we added equal quantities of p53 protein based on western blotting (Figure 6B insert) and normalized binding values from each cell type by setting p21LWT equal to 1. Both DOXO-induced and wt p53 binding spectra are shown in Figure 6A; with binding levels for most REs showing little difference (see also Figure S7A). While the profiles are remarkably similar, given that p53 is derived from two different cell types and two types of stimulus, some probes display differences in binding. The very strong ConA RE (perfect consensus) and the 14-3-3σ RE showed somewhat increased binding with DOXO treatment relative to wt p53, while GADD45, PCNA and MDM2 REs displayed reduced binding. Given the difference between these cell preparations, it is unclear if the binding differences observed are biologically meaningful. While treatment-specific, posttranslational modifications, such as acetylation at lysine 317, phosphorylation at serine 33 or cofactor interactions, have been hypothesized to mediate p53-mediated gene expression and/or binding [1],[2],[32],[33], this may not be the case for some established tumor cell lines [34].

In general for both DOXO-activated and wt p53, we observed that p53 REs from cell cycle arrest genes (p21LT, 1.22; 14-3-3σ, 1.26) and DNA repair genes (p53R2, 1.02; PCNA, 0.33; GADD45, 0.44) showed greater relative binding than REs from apoptotic response genes (PUMA, 0.42; NOXA, 0.12; AIP1, 0.23; BAX-B, 0.07). Importantly, these wild type patterns recapitulate in vivo transactivation experiments using a reporter assay in SaOS2 cells expressing p53. Figure 6A (inset) shows the strong correlation between p53 binding and transactivation capability of p53 REs measured in mammalian cells for several known p53 target gene REs (R^2^ = 0.97, p = 4.39E-05).

Mutations in p53 that lead directly to tumor growth are often described as having a loss- or gain-of-function, as well as dominant-negative phenotype [35],[36]. The basis for these descriptive names is presumed to be structural changes in the protein that lead to functional effects and influence consequent tumor phenotype. As shown in Figure 6B, p53 mutants carrying DNA binding domain changes, R175H and S121F (previously characterized in SaOS2 cells [22]), displayed dramatically attenuated binding compared to wild type p53 (Figure 6A, 6B). However, not all target REs are affected to the same extent. The classical tumor suppressor R175H p53 mutant (grey stippled bar) appears to abolish binding entirely as expected and several other loss-of-function, DNA-binding domain mutants (G245S, C277W, G279E) tested also showed total loss of binding (data not shown). Notably, the change-of-spectrum S121F mutant (open bar) displays significantly reduced binding for most REs compared to wt p53 (mean reduction = 51%) but S121F retains strong binding for several REs, particularly those found upstream of the NOXA, BAX-B, RGC and CycG genes (noted by # symbol). This alteration in binding profile could translate to differences in transactivation and eventual phenotypic consequences [22],[37].

## Discussion

Understanding variation in complex biological responses, such as those mediated by the p53 tumor suppressor, requires integrating bioinformatics with experimental approaches that reveal fundamental molecular events and their phenotypic consequences at the cellular level. The relationship between the degree of DNA binding and target gene transactivation has been the subject of many studies and there is considerable interest in how human genetic variation in TF binding sites may impact gene expression and disease susceptibility [5],[9],[10],[11],[38],[39],[40],[41],[42]. Several recent studies have identified SNPs in p53 REs and evaluated their impact on expression or binding [5],[30]. The present results suggest that MAPD can be useful for assessing the magnitude of p53 binding to newly discovered candidate sequences and also for evaluating the impact of SNPs or mutations in those sequences.

Whole genome association studies frequently identify SNPs in gene regulatory regions containing putative TFBSs; assessing TF binding using the approach outlined here is one practical way to evaluate the effects of regulatory region SNPs. One issue in developing good prediction of TFBSs has been the lack of quantitative binding data. We show that integrating binding information from site-directed mutagenesis of a p53 consensus sequence into a computational binding site model improves prediction for a second set of REs.

We used MAPD to investigate sequence-specific binding between p53 protein and a number of known and well-studied REs in the p53 pathway. Various in vitro assays have suggested that p53 has a higher affinity for REs in cell-cycle arrest and DNA repair genes than the REs in apoptosis genes [4],[24]. Similar observations were obtained in vivo using a yeast-based system [22],[23], and the present results are in agreement. This is also supported by Riley et al, who report that apoptosis-related p53 REs differ more from consensus binding sequence than cell cycle-related REs. In addition an evolutionary analysis [43] indicates that p53 RE sequences in human apoptosis genes tend to diverge considerably from those of rodents—although some weak binding REs do demonstrate strong evolutionary conservation.

We observed a strong correlation between binding to target p53 REs and p53-driven luciferase gene expression in mammalian cells (Figure 6A). We have also seen that binding correlates with expression for the p21 RE when constructed with variable numbers of nucleotides between p53 half-sites [44]. These correlations suggest that our in vitro binding assay using p53 in nuclear extracts may be a surrogate for some in vivo transactivation studies. However, further work to evaluate binding to full length native promoter sequence with direct comparisons to chromatin IP and expression is needed [45],[46]. The importance of p53 binding has been highlighted by Shaked et al al [34] who reported that p53 activation status and RE occupancy differ between tumor cell lines and normal primary cells. Our approach, assessing binding with nuclear extracts, can be used with tumor lines, EBV transformed lymphoblasts or normal primary cells. This will allow quantitative evaluation of the impact of post-translationally modified and activated wt p53 on binding to target gene REs as in Figure 6, or under various stress and DNA-damaging situations as in Shaked et al [34]. Given that p53 mutations are the most common genetic changes in cancer and are a major determinant of treatment outcome [35],[36], the MAPD system may be useful in assessing the impact of mutations on p53 RE interactions (Figure 6B). Since radiation and alkylating agents are primary therapies in cancer treatment, understanding how exposures affect p53 activation and binding could provide insight into tumor biology, carcinogenesis and treatment response [35],[36].

TF binding to REs is a necessary step in regulating transcriptional networks [8]. EMSA and chromatin immunoprecipitation (ChIP) have been essential in advancing the study of gene regulation. However, both approaches are low in sample throughput, labor–intensive, difficult to quantify and have a limited dynamic range. More recently, ChIP combined with microarrays [34] or sequencing, and protein binding arrays [47],[48] have allowed parallel analyses of TF binding to thousands of sequences, and these techniques are highly effective for qualitative discovery of binding sites. However, currently these approaches have experimental constraints, including relatively low sample throughput, uncharacterized variability, and are expensive. Surface plasmon resonance and fluorescence anisotropy are useful for determining detailed biochemical parameters, but like protein binding arrays are currently restricted to purified proteins. MAPD complements existing approaches for the functional examination of candidate binding sequences because this system permits relative quantification and allows multiple endpoints (up to 100 DNA REs per experiment including internal reference controls). In addition, the use of cell extracts in a rapid, low-cost 96-well format enables complex experimental designs with large numbers of samples and replication. An automated format is possible and screening for inhibitors of binding or compounds that selectively restore binding could be accomplished. A strength of the MAPD approach over other binding methods is the inclusion of an internal reference positive control (for example, p21, a strong binding RE) and a negative control (WRNC, a nonbinding sequence) within the multiplexed assay which provide for quality control and normalization across experiments.

MAPD is a general methodology as demonstrated by detection of not only p53 binding, but also the nuclear hormone receptor ERα binding its RE (Figure S5). MAPD could be adapted to detect the impact of multiple proteins in a transcription complex or to evaluate multiple binding sites within native or artificially constructed promoters. Ongoing studies are examining the functional evolution of p53 response elements across mammalian species [43],[49], co-evolution of the p53 protein, as well as rules for p53 transactivation [50]. Because of MAPD's sensitivity and dynamic range, the impact of SNPs or mutations on binding can be assessed providing a valuable tool in genomics research. We found that subtle changes in binding sequence can have effects that are not captured with existing computational models. Using nuclear extracts from human cells expressing p53 mutants, R175H and S121F, we observe a loss of binding and a change of binding specificity, respectively, consistent with their effect on gene expression. The correlation between binding in this nuclear extract-based in vitro system and transactivation in mammalian cells suggests the important role that sequence-specific p53 interactions play in transcriptional activation. MAPD will facilitate studies of how variation in target binding sites affects binding and fine-tuning the regulation of transcriptional networks as well as the impact of polymorphic variation on master regulatory networks.

## Materials and Methods

### Cell Culture and Treatments

Human lymphoblastoid cells were grown in RPMI 1640 media supplemented with 15% heat-inactivated fetal bovine serum (Invitrogen Corp., Carlsbad, CA) and incubated at 5% CO_2_ at 37°C with 1% penicillin-streptomycin antibiotics (Invitrogen). The cell lines GM12824 and GM12825 used for exposure were purchased from Coriell Cell Repositories (Camden, NJ). Cells were grown to ∼8.5×10^5^ cells/mL before being exposed to 0.6 µg/mL (1 mM) doxorubicin (Sigma, St. Louis, MO) for 18 hours at 37°C. Human osteosarcoma cell lines SaOS-2 (HTB-85, ATCC, Manassas, VA) and U2OS (HTB-96, ATCC) were grown in McCoy's A5 medium supplemented with 10% FBS and 100 µg/ml of penicillin and streptomycin. Cells were incubated at 37°C with 5% CO_2_.

### Transient Transfection

For transient transfections, 1×10^6^ SaOS2 cells were seeded in a 100 mm plate. After 24 hours cells were transfected with 2 µg of purified endotoxin-free p53 expression vectors in the presence of non-liposomal transfection reagent, FuGENE 6 (Roche Diagnostics, Indianapolis, IN) according to manufacturer's protocol. U2OS cells were transfected with 2 µg pSG5-ERα expression vector, provided by Dr. Gilbert Schonfelder, 24 hours prior to preparation of extracts. Plasmids pC53-SN3[51] coding for human p53 cDNA under the control of CMV promoter and pCMV-Neo-Bam were provided by Dr. Bert Vogelstein. p53 mutants were made by site-directed mutagenesis according to the manufacturer's protocol (QuickChange Site-Directed Mutagenesis kit, Stratagene, La Jolla, CA). p53 constructs were confirmed by sequencing.

### Nuclear Protein Extraction and Quantification

The presence of p53 in extracts was assessed by western blot and TransAM p53 Transcription Factor assay (Active Motif). Nuclear protein was extracted from non-treated and doxorubicin-treated cells according to manufacturer's protocol (Active Motif, Carlsbad, CA) for ∼8.8×10^6^ cells with the following modification. Cells were transferred to 15 mL centrifuge tubes, spun at 4°C (200×g, 5 min) and washed in 1× PBS/phosphatase. Protein concentration was measured in triplicate using the BCA Protein Assay kit (Pierce Biochemical, Rockford, IL), followed by a fluorescence plate read using a HTS 7000 Bio Assay Reader (Perkin Elmer, Waltham, MA).

### Western Blot Analysis

Equal amounts of whole cell or nuclear extracts were separated on NuPAGE BisTris gels and transferred to polyvinylidene difluoride membranes (Invitrogen). The blots were probed with primary antibodies (Santa Cruz Biotechnology, Inc., Santa Cruz, CA) for p53 (PAb1801 and DO-1) and actin (C-11). For ERα detection we used rabbit polyclonal anti-ERα (H-184, Santa Cruz). Bands were detected using horseradish peroxide-conjugated secondary antibodies (Santa Cruz) and the ECL Western Blotting System (Amersham Biosciences, Piscataway, NJ).

### General Oligonucleotide Design

The double-stranded DNA fragments incorporated a forward oligonucleotide with a 24-nt “tag” at the 5′ end linked to a 50-nt fragment containing a sequence of interest. The forward oligonucleotide was hybridized to a unique 24-nt “anti-tag” present on each FlexMAP bead type (Luminex, Austin, TX). The 50-nt modular fragment typically contained a 20-nt RE in the middle of a 30-nt common backbone sequence (sequences are described in Supporting Information). For p53 REs with a 1-nt spacer (MDM2RE2, IGFBP3 and BAXA), the backbone length was decreased to 29-nt. For ERα REs (17-nt in length), the backbone sequence was increased to 33-nt. Reverse oligonucleotides complementary to the 50-nt modular fragment were synthesized with a 5′ biotin moiety. All oligonucleotides were synthesized by Invitrogen and are listed in the Supporting tables.

### Preparation of Oligonucleotide-Bead Targets

We used 15 µL each bead type (approximately 2,500 beads) per reaction. Beads were vortexed for 30 seconds and added to double-stranded DNA containing the corresponding tag sequence in an annealing buffer containing 5 mM Tris (pH 7.5), 10 mM NaCl, and 0.5 mM EDTA. The final primer concentration was 1.5 µM. Annealing was performed in a PCR cycler (95°C for 5 min, followed by an incremental 5°C decrease, holding for 1 minute at each temperature, until reaching 45°C). Beads were then placed in a filter plate (MultiScreenHTS-BV filter plate, Millipore Corp., Bedford, MA) and washed several times with Wash Buffer 1 (Assay Buffer A, Cytokine Reagent Kit, Bio-Rad Laboratories, Hercules, CA).

### Microsphere Protein-DNA Binding

Washed beads with conjugated DNA were then resuspended in the appropriate volume of protein binding buffer (0.05 M KCl, 0.1 mM EDTA, 5 mM DDT, 0.05% Triton X-100, 10 mM MgCl_2_, 1.0 mg/mL BSA, 0.5 mM ATP and 25 mM HEPES, pH 7.6) supplemented with 150 pmoles non-competing double-stranded oligo, WRNC. The noncompeting oligo from the TransAm p53 kit (Active Motif, Carlbad, CA) was also effective. In a typical binding assay (detection of p53 and ERα), beads were resuspended in 50 µL protein binding buffer and supplemented with 1.75 µg nuclear extracts from either treated or non-treated cells, followed by thorough mixing for 15 seconds. Beads were incubated with nuclear extracts for 1 hour at room temperature in the dark, transferred onto a filter plate, washed 3 times with 50 µL Wash Buffer 2 and incubated for 30 min with primary antibody. For p53 detection, we used mouse monoclonal anti-p53 primary antibody (1∶500 dilution, clone DO-7, BD Biosciences, San Jose, CA). For ERα detection, we used rabbit polyclonal anti-ERα (1∶500 H-184, Santa Cruz). Beads were washed 3 times with 50 µL Wash Buffer 2 (50 mM KCl, 0.1 mM EDTA, 5 mM DTT, 0.5 mg/mL BSA, 0.05% Triton X-100, 10 mM MgCl_2_ and 25 mM HEPES, pH 7.6). The beads were then incubated with phycoerythrin-conjugated secondary antibodies (1∶75 dilution, R-phycoerythrin-conjugated goat anti-mouse (p53) or 1∶75 dilution, R-phycoerythrin-conjugated goat anti-rabbit (both purchased from Invitrogen) for 30 min and washed 3 times with 50 µL Wash Buffer 2. Dilutions were prepared using a buffer made up of PBS (5 mM Phosphate, 1.35 mM KCl, 68 mM NaCl) supplemented with 0.5% Tween 20 and 1% BSA. For signal normalization, DNA-conjugated beads were treated separately with phycoerythrin-conjugated streptavidin, 1× streptavidin-PE diluted in Wash Buffer 1 for 20 min in the absence of any nuclear extracts, then washed 3 times with 50 µL Wash Buffer 2. For signal detection, beads were then re-suspended in 150 µL Wash Buffer 1 and subjected to flow analysis using a Bio-Plex 200 (Bio-Rad Laboratories) by following the manufacturer's recommendation for instrument settings and calibration. All binding reactions were conducted in triplicate.

### Measurement of p53 Binding to SNP Alleles

The effect of SNPs on p53 binding to putative REs was examined using the microsphere assay for protein-DNA binding as described above. Each bead type was conjugated to double-stranded oligonucleotides that contained either the predicted strong or weak allele in separate annealing reactions (*i.e.*, ADARB1 Strong and ADARB1 Weak were conjugated to bead 12). Multiplexed allele sets were tested in parallel binding reactions. To assess p53 binding, beads were incubated with nuclear extracts derived from human either human lymphoblastoid (Figure 5) or U2OS cells (Figure S8) that were treated with doxorubicin or untreated (not shown). Negative and positive control oligonucleotide-bead targets were examined in each parallel reaction, and untreated nuclear extracts were used as an additional negative control.

### Bead-Specific Corrections

Oligonucleotide density on beads varies among bead types[14],[52]. Therefore, bead-specific contribution to overall signal must be taken into account. To account for bead-specific effects, we used a 5′ biotin moiety on the reverse oligonucleotide bound to a phycoerythrin-conjugated streptavidin to measure the maximal signal from each bead type used. For each bead type, the protein-binding signal was normalized by the bead-specific signal, independent of nuclear extracts and reported as relative binding. In addition, FlexMap bead tag/anti-tag oligonucleotide sequences should be evaluated for their similarity to target sequences and binding to protein of interest.

### Bioinformatics

We implemented a position weight matrix (PWM) for quantitatively estimating DNA-protein binding and for predicting novel REs [40]. In a standard PWM model, entries are the probability of observing nucleotides at each position for the set of sequences examined. The p53 RE PWM was computed from 59 published p53 REs (118 halfsites) (Table S2) [43]. The PWM score for a p53 RE was calculated by scoring each half-site independently, then summing the two. The ERα PWM was based on 28 published ERα REs (Table S1). The binding PWM and sequence logo was calculated by modifying the approach of Veprintsev and Fersht [30]. Briefly, a binding difference matrix was generated by subtracting the binding value for each of 60 ConA variants from the ConA binding value. Equations 2, A.1, A.2 from the Appendix in Veprintsev and Fersht [30] were used to generate a binding-based information content (IC) position weight matrix. This IC PWM was used to create the sequence logo shown in Figure 4B using the enoLOGOS software (http://biodev.hgen.pitt.edu/cgi-bin/enologos/enologos.cgi), and was used to calculate the predicted binding scores for the RE sequences plotted in Figures 4D. These REs plotted in Figure 4D are the same as those in Figure 6 and the sequences are listed in Table S1.

## Supporting Information

Figure S1 Microsphere assay procedure. A) Double stranded oligonucleotides containing sequences of interest are hybridized to FlexMap beads and multiplex groups of beads are prepared. B) Beads are incubated with nuclear extracts containing transcription factor. C) Primary and secondary antibodies are added. D) Beads are sorted and fluorescence signal is quantified for each bead type. A critical technical feature of the assay is the use of FlexMAP oligonucleotide-coated microspheres which allow for the attachment of desired sequences to the bead. However, because the density of oligonucleotide tags present on the surface of the beads varies between bead types and bead batches, a bead type-specific signal correction must be made in each experiment [14],[52]. Each double-stranded oligonucleotide tested had a biotin incorporated at the free 5′end (not shown in all drawings) and following the hybridization, was conjugated with streptavidin-phycoerythrin and read on the Bioplex 200 instrument. These biotin-streptavidin-phycoerythrin values provide a readout for how many double-stranded target oligonucleotides are present on the surface of each bead type, and were used to normalize bead signals in each experiment.
(0.07 MB PDF)

Figure S2 Tests of system components. A multiplex set of six oligonucleotide-conjugated beads, each carrying a p53 binding site (ConA, ConC, p21, PUMA, GADD45) and a negative control (WRNC), was incubated under various conditions as shown and analyzed for p53 binding as described in Supplemental Materials and Methods. Lanes 1–5,7 and 8 show background and non-specific interaction. A modest signal is detected following incubation for 60 minutes with nuclear extracts from untreated lymphoblastoid cells in the presence of primary and secondary antibodies (lane 6). This signal was slightly enhanced in the presence of free noncompeting oligo (lane 10) and attenuated in the presence of a competing oligo (lane 11). A strong signal was detected (lane 9) following incubation for 60 minutes with nuclear extracts from lymphoblastoid cells treated with Doxo (30 mg/ml for 18 hrs). In addition the signal was strongly enhanced in the presence of free NC oligo (lane 12) for all p53 REs present in the 6-plex. The increased signal in the presences of noncompeting oligonucleotide is likely due to blocking of nonspecific DNA-binding proteins in the extract thereby enhancing specific p53 binding to target oligonucleotides. The signal was greatly attenuated in the presence of 150 pmol competing oligonucleotide (lane 13). The minimal signal for lane 13 indicates there is little if any nonspecific binding of p53 to beads or antitag sequences, even in the absence of noncompeting oligonucleotides. Note the two scales of vertical axis. Values shown are mean for each bead type+/−SD (n = 3).
(0.77 MB PDF)

Figure S3 p53 binding in the presence of non-competing and competing oligonucleotides. Specificity was ascertained by varying amounts of competing oligonucleotide which was observed to reduce specific binding in a concentration-dependent manner. Similarly, the use of a noncompeting (or nonspecific) oligonucleotide, which blocks binding of nonspecific DNA binding proteins to the target sequences, enhances the sensitivity as well as detection limit and dynamic range of the assay. A multiplex set of 6 oligonucleotide-conjugated beads, 5 of which carry p53 REs (ConA, ConC, P21, PUMA, GADD45) and a negative control (WRNC), were incubated with nuclear extracts (NT: non-treated; Doxo: Doxorubicin-treated) for 60 minutes in the presence of variable amounts of either non-competing (NC) or competing (Comp) oligonucleotides and analyzed for p53 binding (see Materials and Methods for sequence of oligonucleotides used). A) The oligonucleotide set was incubated with decreasing amounts of non-competing oligonucleotide (shown in pmoles per reaction) and 1.75 micrograms of nuclear extracts from Doxo-treated cells. B) Treatment with decreasing amounts of a competing oligonucleotide and 1.75 micrograms of nuclear extract from Doxo-treated cells. NC oligonucleotides were not added in this experiment. Under these conditions ∼1 pmole of competing oligonucleotide is needed to eliminate p53 binding. The relative binding intensity (value shown on vertical axes) was obtained for each oligonucleotide as discussed in Materials and Methods. Note breaks in the scale of vertical axes. Values shown are means for each bead type±SD (n = 3).
(0.06 MB PDF)

Figure S4 Concentration versus binding isotherm for nuclear extracts containing activated p53. A multiplex set of 6 oligonucleotide-conjugated beads, five carrying p53 REs (ConA, ConC, P21, PUMA, GADD45) and one a negative control (WRNC), was incubated with variable amounts of DOXO treated nuclear extracts in the presence of 150 pmoles of non-competing oligonucleotides for 60 minutes and analyzed for p53 binding. A) Normalized binding for each oligonucleotide is shown with varying amounts of nuclear extracts from Doxo-treated cells. The relative binding intensity (value shown on vertical axes) was obtained for each oligonucleotide as discussed in Materials and Methods. Values shown are means for each bead type±SD (n = 3). Nonlinear regression was used to determine BMax and Kd. A concentration of nuclear extract that was saturating for the p21 RE (1.75 ug) was chosen for the assay. An additional parameter investigated included the impact of bead quantity on relative binding. Bead quantity was varied from 2500 to 40,000 beads (representing up to 80 bead types), each bead represented at equal concentration. These experiments were carried out under varying protein concentrations. Using 1.75 ug protein in the hybridization, no effect of bead number on binding was observed. At lower protein concentrations (less than 1.2 ug protein) binding values were reduced for higher bead quantities in a linear, bead-quantity dependent manner. Thus protein and bead/probe number need to be optimized for a given system.
(0.05 MB PDF)

Figure S5 Detection of the interaction between ERα and its cognate REs. An oligonucleotide set representing 8 previously validated ERα REs (see supplementary materials for complete sequences) was assembled and tested for ERα binding. The panel included ConA and WRNC (from the p53 RE set). A) The ERα RE panel was treated with nuclear extracts from cells with no estrogen receptor (Lymph NT, lymphoblasts; Lymph Doxo, Doxorubicin treated; U2OS NT, untransfected U2OS cells) or pSG5-ERα-transfected U2OS cells (U2OS+pSG5-ERα). ER α interaction with ERE sequences was assessed by treating with anti-ERα antibody. Bar values are means for each bead type+/−SD (n = 3). The relative binding intensity (value shown on vertical axes) was obtained for each oligonucleotide as discussed in Materials and Methods. Nuclear extracts from cells that do not express ERα (lymphocytes, Dox-treated lymphocytes and U2OS cells) produced very low signal. In contrast, ERα binding to bona fide EREs was strongly detected following incubation with extracts from an ERα over-expressing cell line (U2OS−pSG5-ERα). B) Experimental binding vs calculated PWM for ERα. Comparison of experimental ERα binding (relative binding, n = 3) vs calculated ERα binding values (PWM). EBAG9, EFP, and COX7RP probes contain ERα binding sites closely matching the ERE consensus and displayed the highest level of binding to ERα. TERT, pS2, LTF and KRT19 contain one or two changes from consensus and showed moderate ERα binding, while CTSD had the lowest binding signal. Values shown are means for each bead type±SD (n = 3). Within this small set of EREs, the binding was highly correlated (R^2^ = 0.78, p<0.001) with their sequence match to consensus (based on calculated PWM values). For instance, EBAG9, EFP, and COX7RP contain ERα binding sites closely matching the ERE consensus and displayed the highest level of binding to ERα. TERT, pS2, LTF and KRT19 contain one or two changes from consensus and showed moderate ERα binding, while CTSD had the lowest binding signal. Unexpectedly, ERα binding was observed for the p53 positive and negative control sequences ConA and WRNC. Further evaluation using an ERE PWM model revealed that these sequences contain ERE-like motifs that score as high as CTSD target sequences. This assay requires further optimization.
(0.05 MB PDF)

Figure S6 Multiplex capability of MAPD using extracts from untreated and treated cells. A total of 60 oligonucleotides, each bearing a single nucleotide variation of a perfect p53 consensus binding site (ConA = GGGCATGTCCGGGCATGTCC) was generated. ConA sequence is shown in large letters. The CATG core of each half-site is shown in a right-facing bracket. Systematic base substitutions at all positions within ConA are shown in small letters. For instance, 1G>C is an RE with the sequence CGGCATGTCCGGGCATGTCC. In addition, a positive control bead (ConA RE) and a negative control bead (WRNC: lacks a p53 binding RE) were also generated. Each oligonucleotide variant was coated onto a unique microsphere type. Equal amounts of each microsphere type were mixed to generate a multiplex of 62 types of microspheres. Up to 82 oligonucleotides have been successfully multiplexed (not shown). Beads were incubated with nuclear extracts from untreated cells (NT, checkered bar) or nuclear extracts from doxo-activated cell (Doxo, black bars). Data from Doxo treated cells in this figure are also plotted in Figure 3b. The relative binding intensity (value shown on upper horizontal axis) was obtained for each oligonucleotide (white bar) as discussed in Materials and Methods. Bar values are means for each bead type±SD (n = 3).
(0.67 MB PDF)

Figure S7 Comparative binding. A) Normalized binding values from Doxo-induced p53 binding for p53 REs shown in Figure 6 are plotted against binding values for Wt p53 expressed in SaOS-2 cells (data from Figure 6A). Binding values between the two extracts are highly correlated, linear regression line is plotted, r^2^ = 0.86. B) Normalized binding values (n = 8) from Doxo-induced p53 binding for p53 REs shown in Figure 4C are plotted against available ChIP-PET-SAGE values from Wei et al [31] downloaded from the UCSC browser. A correlation coefficient of r^2^ = 0.58 (p = 0.01) was observed for the comparison of MAPD-determined relative binding vs ChIP-PET-SAGE for 8 known p53 REs despite using different cell lines, treatments, and methods.
(0.04 MB PDF)

Figure S8 Evaluation of eight p53 RE SNPs using nuclear extracts containing activated p53 from DOXO treated U2OS cells (same procedure as Figure 5).
(0.04 MB PDF)

Table S1 Oligonucleotide sequences.
(0.07 MB DOC)

Table S2 p53 response elements used to build position weight matrix.
(0.10 MB DOC)
