# Supplementary material for: Probing the Functional Impact of Sequence Variation on p53-DNA Interactions Using a Novel Microsphere Assay for Protein-DNA Binding with Human Cell Extracts
Source: PLoS Genet. 2009 May 8;5(5):e1000462. doi: 10.1371/journal.pgen.1000462 (PMC2667269; doi:10.1371/journal.pgen.1000462)

A. Create unique bead-sequence combinations

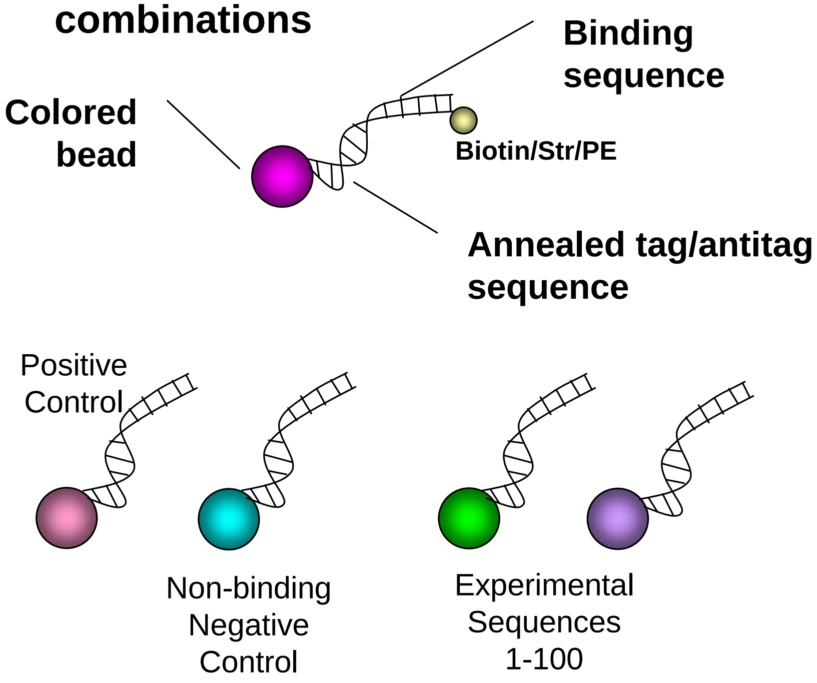

B. Incubate beads with nuclear extract

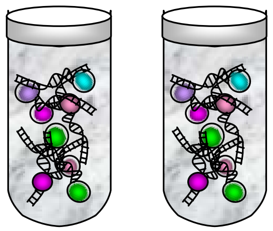

C. Detect transcription factor

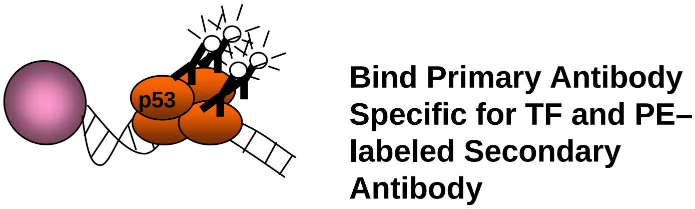

D. Flow sort beads to detect bead identity and TF signal

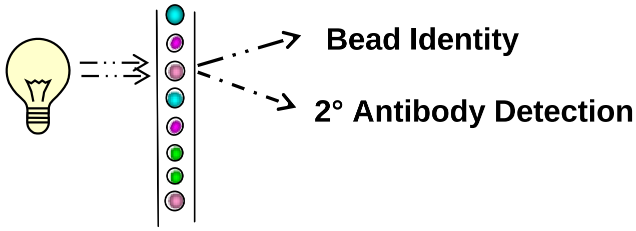

Supplement: Figure S1 — Microsphere assay procedure. A) Double stranded oligonucleotides containing sequences of interest are hybridized to FlexMap beads and multiplex groups of beads are prepared. B) Beads are incubated with nuclear extracts containing transcription factor. C) Primary and secondary antibodies are added. D) Beads are sorted and fluorescence signal is quantified for each bead type. A critical technical feature of the assay is the use of FlexMAP oligonucleotide-coated microspheres which allow for the attachment of desired sequences to the bead. However, because the density of oligonucleotide tags present on the surface of the beads varies between bead types and bead batches, a bead type-specific signal correction must be made in each experiment [14],[52]. Each double-stranded oligonucleotide tested had a biotin incorporated at the free 5′end (not shown in all drawings) and following the hybridization, was conjugated with streptavidin-phycoerythrin and read on the Bioplex 200 instrument. These biotin-streptavidin-phycoerythrin values provide a readout for how many double-stranded target oligonucleotides are present on the surface of each bead type, and were used to normalize bead signals in each experiment. (0.07 MB PDF) [file pgen.1000462.s001.pdf]
