# Supplementary figures and images for: Probing the Functional Impact of Sequence Variation on p53-DNA Interactions Using a Novel Microsphere Assay for Protein-DNA Binding with Human Cell Extracts
Source: PLoS Genet. 2009 May 8;5(5):e1000462. doi: 10.1371/journal.pgen.1000462 (PMC2667269; doi:10.1371/journal.pgen.1000462)

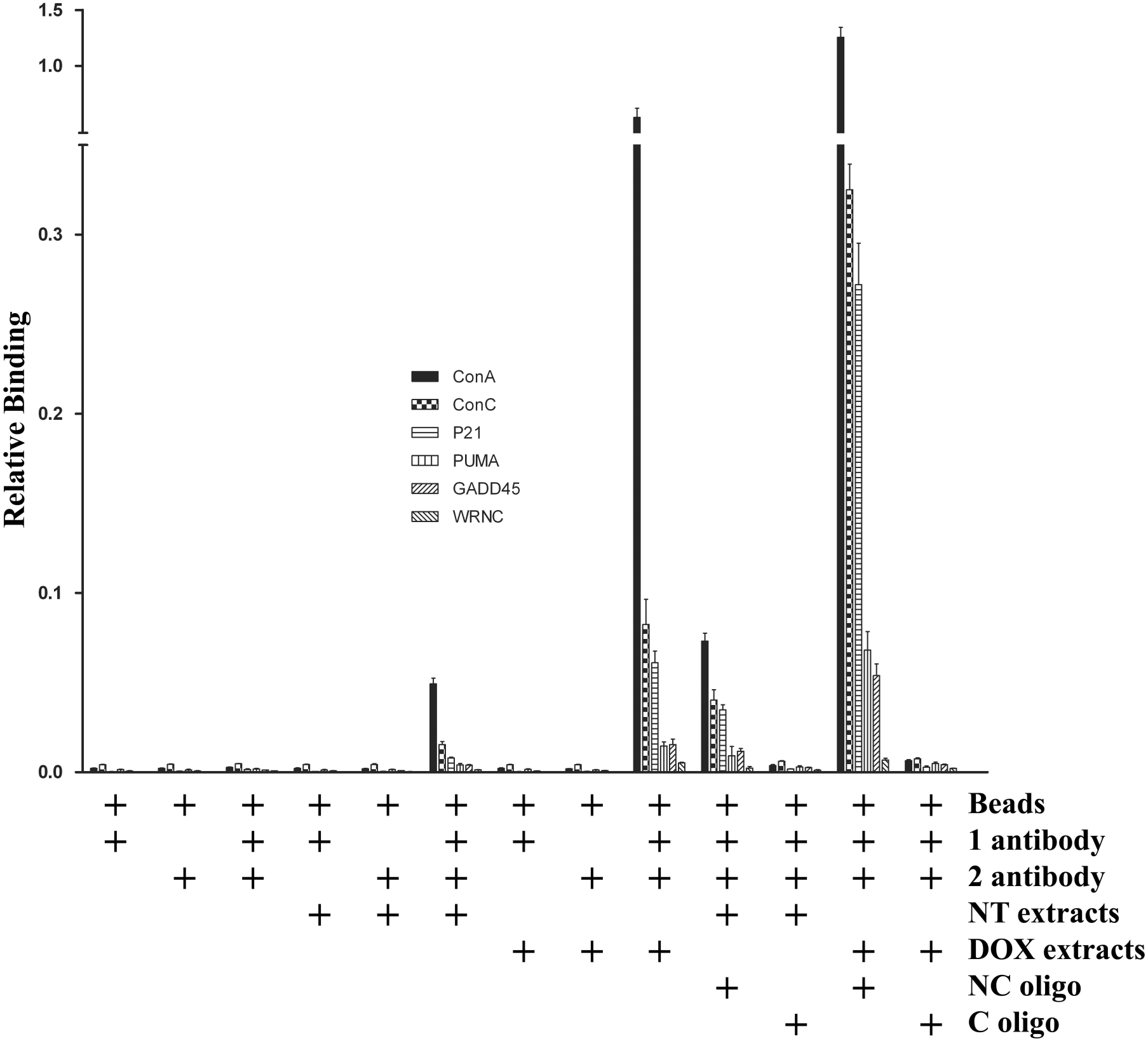

Supplement: Figure S2 — Tests of system components. A multiplex set of six oligonucleotide-conjugated beads, each carrying a p53 binding site (ConA, ConC, p21, PUMA, GADD45) and a negative control (WRNC), was incubated under various conditions as shown and analyzed for p53 binding as described in Supplemental Materials and Methods. Lanes 1–5,7 and 8 show background and non-specific interaction. A modest signal is detected following incubation for 60 minutes with nuclear extracts from untreated lymphoblastoid cells in the presence of primary and secondary antibodies (lane 6). This signal was slightly enhanced in the presence of free noncompeting oligo (lane 10) and attenuated in the presence of a competing oligo (lane 11). A strong signal was detected (lane 9) following incubation for 60 minutes with nuclear extracts from lymphoblastoid cells treated with Doxo (30 mg/ml for 18 hrs). In addition the signal was strongly enhanced in the presence of free NC oligo (lane 12) for all p53 REs present in the 6-plex. The increased signal in the presences of noncompeting oligonucleotide is likely due to blocking of nonspecific DNA-binding proteins in the extract thereby enhancing specific p53 binding to target oligonucleotides. The signal was greatly attenuated in the presence of 150 pmol competing oligonucleotide (lane 13). The minimal signal for lane 13 indicates there is little if any nonspecific binding of p53 to beads or antitag sequences, even in the absence of noncompeting oligonucleotides. Note the two scales of vertical axis. Values shown are mean for each bead type+/−SD (n = 3). (0.77 MB PDF) [file pgen.1000462.s002.pdf]

# A ER Alpha Binding to EREs

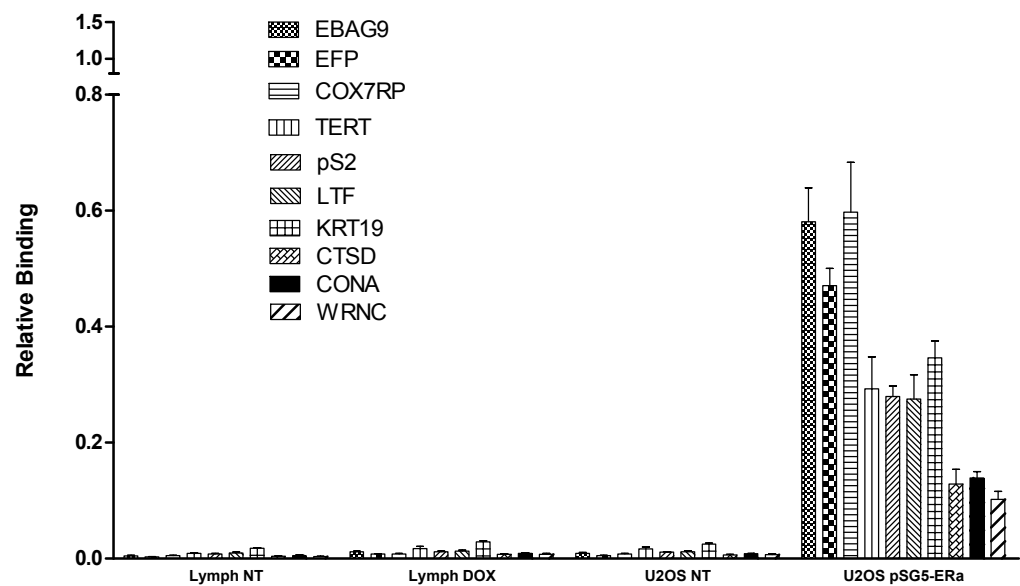

# B Binding vs PWM

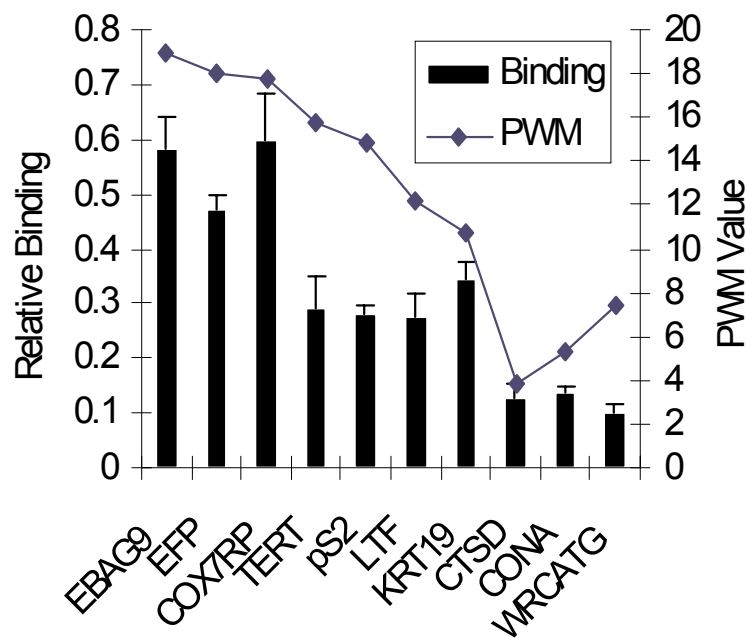

Supplement: Figure S5 — Detection of the interaction between ERα and its cognate REs. An oligonucleotide set representing 8 previously validated ERα REs (see supplementary materials for complete sequences) was assembled and tested for ERα binding. The panel included ConA and WRNC (from the p53 RE set). A) The ERα RE panel was treated with nuclear extracts from cells with no estrogen receptor (Lymph NT, lymphoblasts; Lymph Doxo, Doxorubicin treated; U2OS NT, untransfected U2OS cells) or pSG5-ERα-transfected U2OS cells (U2OS+pSG5-ERα). ER α interaction with ERE sequences was assessed by treating with anti-ERα antibody. Bar values are means for each bead type+/−SD (n = 3). The relative binding intensity (value shown on vertical axes) was obtained for each oligonucleotide as discussed in Materials and Methods. Nuclear extracts from cells that do not express ERα (lymphocytes, Dox-treated lymphocytes and U2OS cells) produced very low signal. In contrast, ERα binding to bona fide EREs was strongly detected following incubation with extracts from an ERα over-expressing cell line (U2OS−pSG5-ERα). B) Experimental binding vs calculated PWM for ERα. Comparison of experimental ERα binding (relative binding, n = 3) vs calculated ERα binding values (PWM). EBAG9, EFP, and COX7RP probes contain ERα binding sites closely matching the ERE consensus and displayed the highest level of binding to ERα. TERT, pS2, LTF and KRT19 contain one or two changes from consensus and showed moderate ERα binding, while CTSD had the lowest binding signal. Values shown are means for each bead type±SD (n = 3). Within this small set of EREs, the binding was highly correlated (R2 = 0.78, p<0.001) with their sequence match to consensus (based on calculated PWM values). For instance, EBAG9, EFP, and COX7RP contain ERα binding sites closely matching the ERE consensus and displayed the highest level of binding to ERα. TERT, pS2, LTF and KRT19 contain one or two changes from consensus and showed moderate ERα binding, while CTSD had th [file pgen.1000462.s005.pdf]

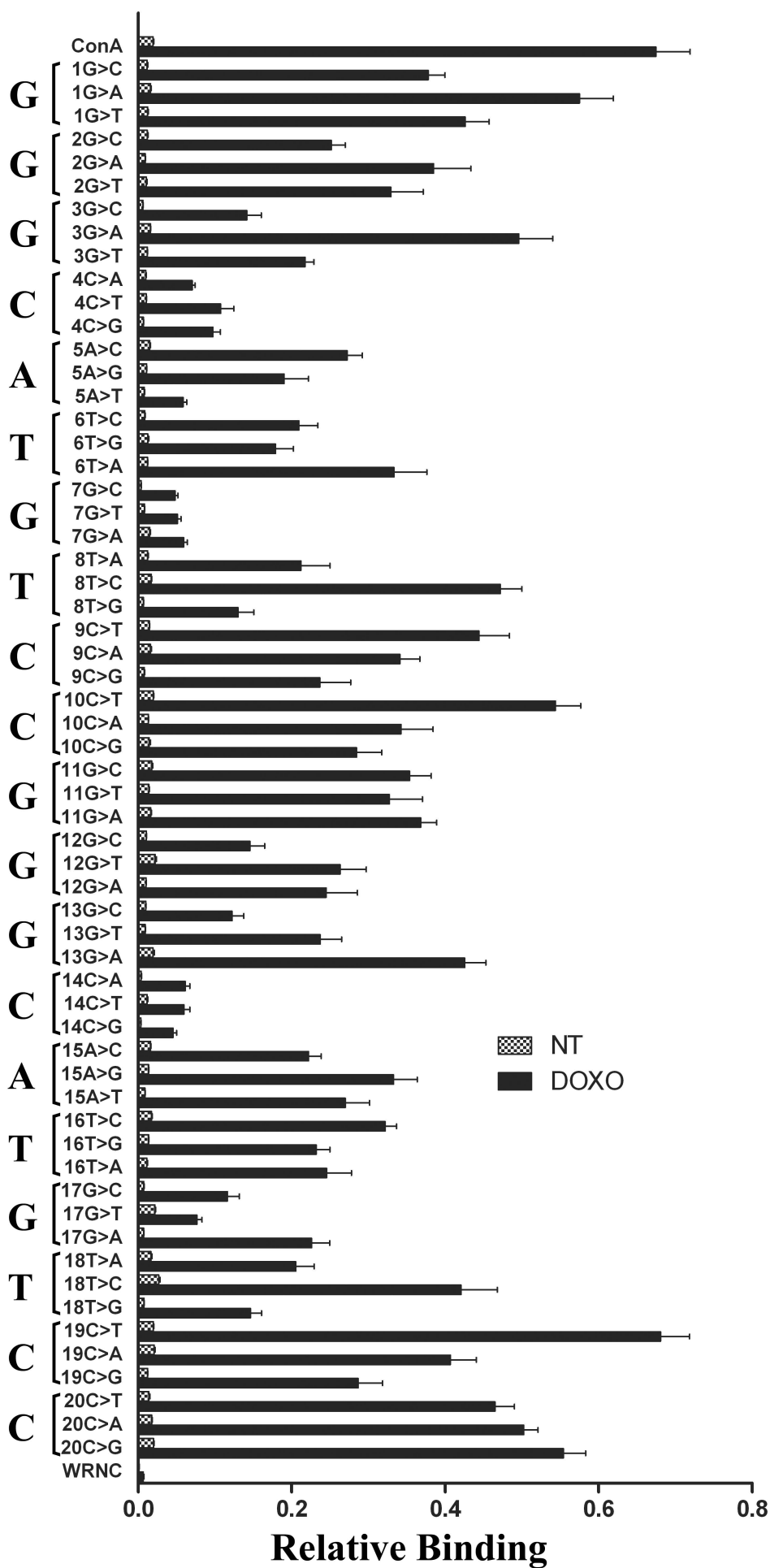

Supplement: Figure S6 — Multiplex capability of MAPD using extracts from untreated and treated cells. A total of 60 oligonucleotides, each bearing a single nucleotide variation of a perfect p53 consensus binding site (ConA = GGGCATGTCCGGGCATGTCC) was generated. ConA sequence is shown in large letters. The CATG core of each half-site is shown in a right-facing bracket. Systematic base substitutions at all positions within ConA are shown in small letters. For instance, 1G>C is an RE with the sequence CGGCATGTCCGGGCATGTCC. In addition, a positive control bead (ConA RE) and a negative control bead (WRNC: lacks a p53 binding RE) were also generated. Each oligonucleotide variant was coated onto a unique microsphere type. Equal amounts of each microsphere type were mixed to generate a multiplex of 62 types of microspheres. Up to 82 oligonucleotides have been successfully multiplexed (not shown). Beads were incubated with nuclear extracts from untreated cells (NT, checkered bar) or nuclear extracts from doxo-activated cell (Doxo, black bars). Data from Doxo treated cells in this figure are also plotted in Figure 3b. The relative binding intensity (value shown on upper horizontal axis) was obtained for each oligonucleotide (white bar) as discussed in Materials and Methods. Bar values are means for each bead type±SD (n = 3). (0.67 MB PDF) [file pgen.1000462.s006.pdf]

A Comparative p53 Binding  
(DOXO vs Wt)

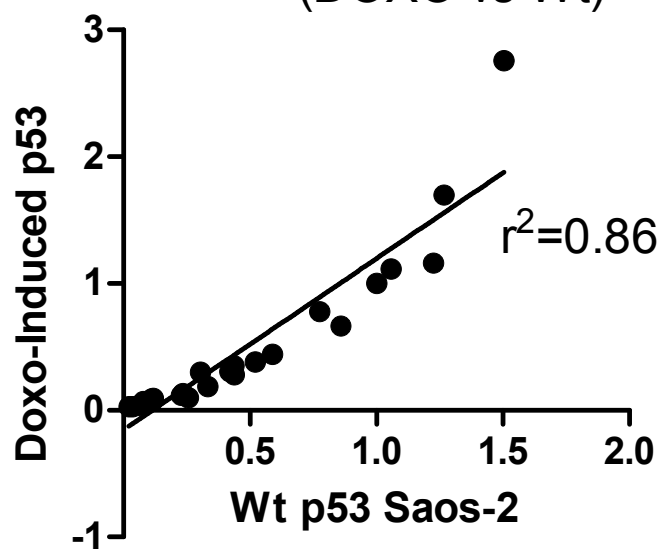

B Binding vs PET- ChIP

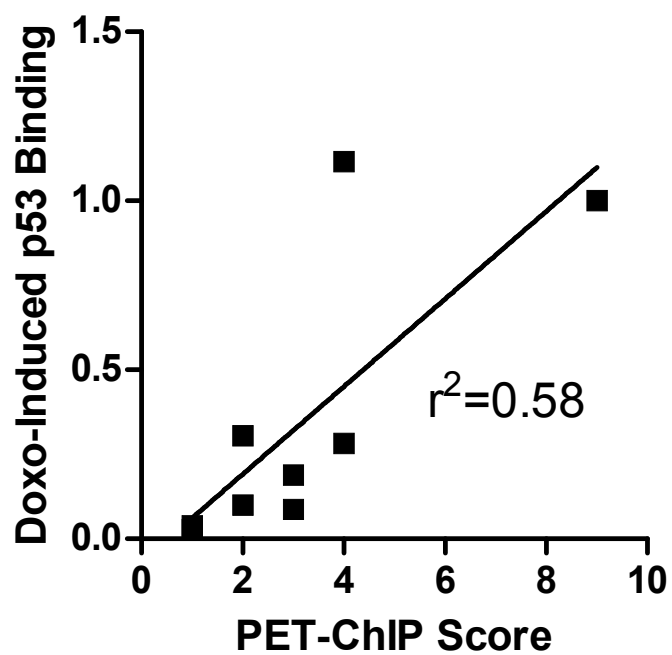

Supplement: Figure S7 — Comparative binding. A) Normalized binding values from Doxo-induced p53 binding for p53 REs shown in Figure 6 are plotted against binding values for Wt p53 expressed in SaOS-2 cells (data from Figure 6A). Binding values between the two extracts are highly correlated, linear regression line is plotted, r2 = 0.86. B) Normalized binding values (n = 8) from Doxo-induced p53 binding for p53 REs shown in Figure 4C are plotted against available ChIP-PET-SAGE values from Wei et al [31] downloaded from the UCSC browser. A correlation coefficient of r2 = 0.58 (p = 0.01) was observed for the comparison of MAPD-determined relative binding vs ChIP-PET-SAGE for 8 known p53 REs despite using different cell lines, treatments, and methods. (0.04 MB PDF) [file pgen.1000462.s007.pdf]

### p53 Binding to SNP Alleles (U2OS Nuclear Extracts)

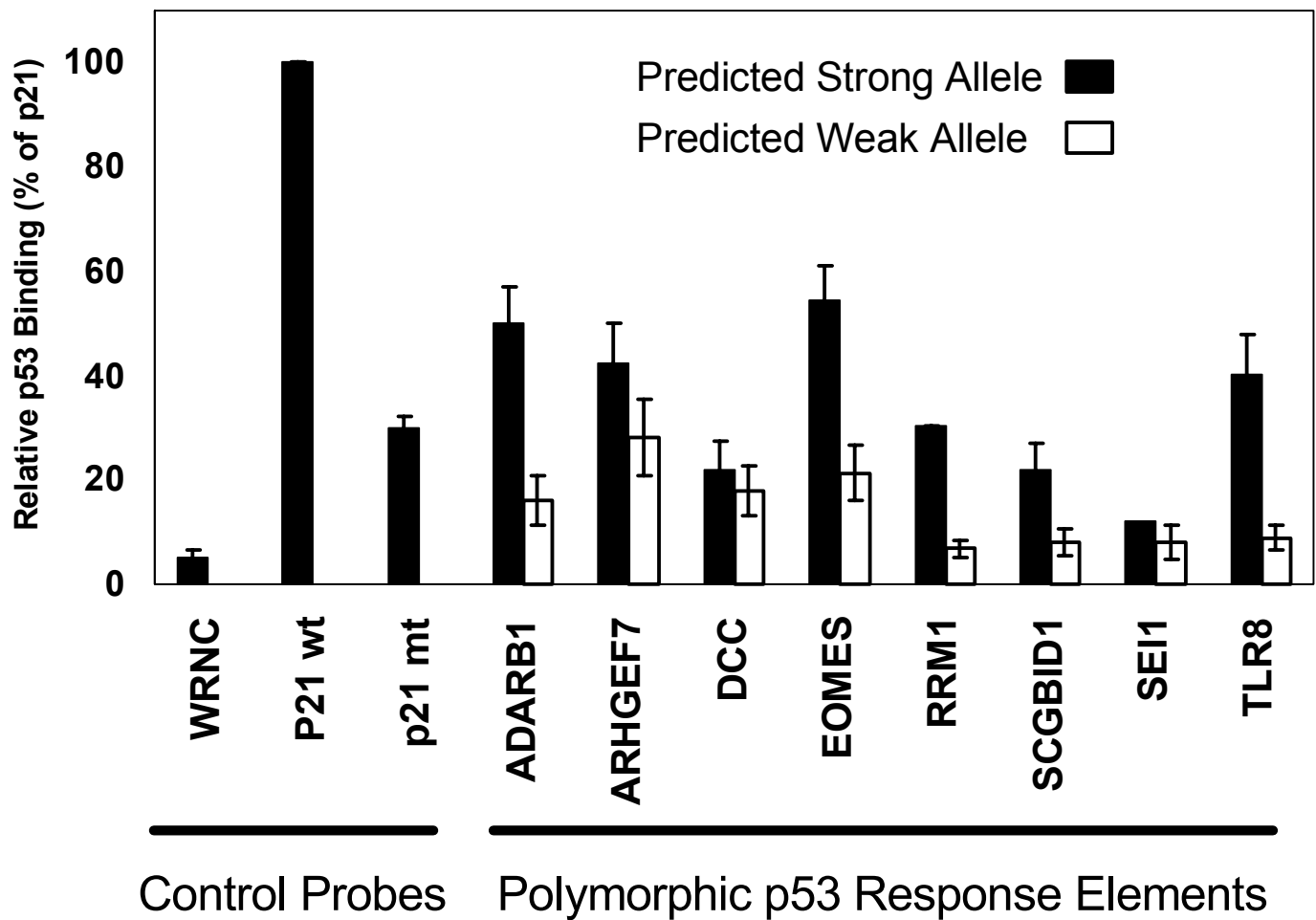

Supplement: Figure S8 — Evaluation of eight p53 RE SNPs using nuclear extracts containing activated p53 from DOXO treated U2OS cells (same procedure as Figure 5). (0.04 MB PDF) [file pgen.1000462.s008.pdf]
