# Supplementary material for: Probing the Functional Impact of Sequence Variation on p53-DNA Interactions Using a Novel Microsphere Assay for Protein-DNA Binding with Human Cell Extracts
Source: PLoS Genet. 2009 May 8;5(5):e1000462. doi: 10.1371/journal.pgen.1000462 (PMC2667269; doi:10.1371/journal.pgen.1000462)

A NonCompeting Oligonucleotide Enhances Binding

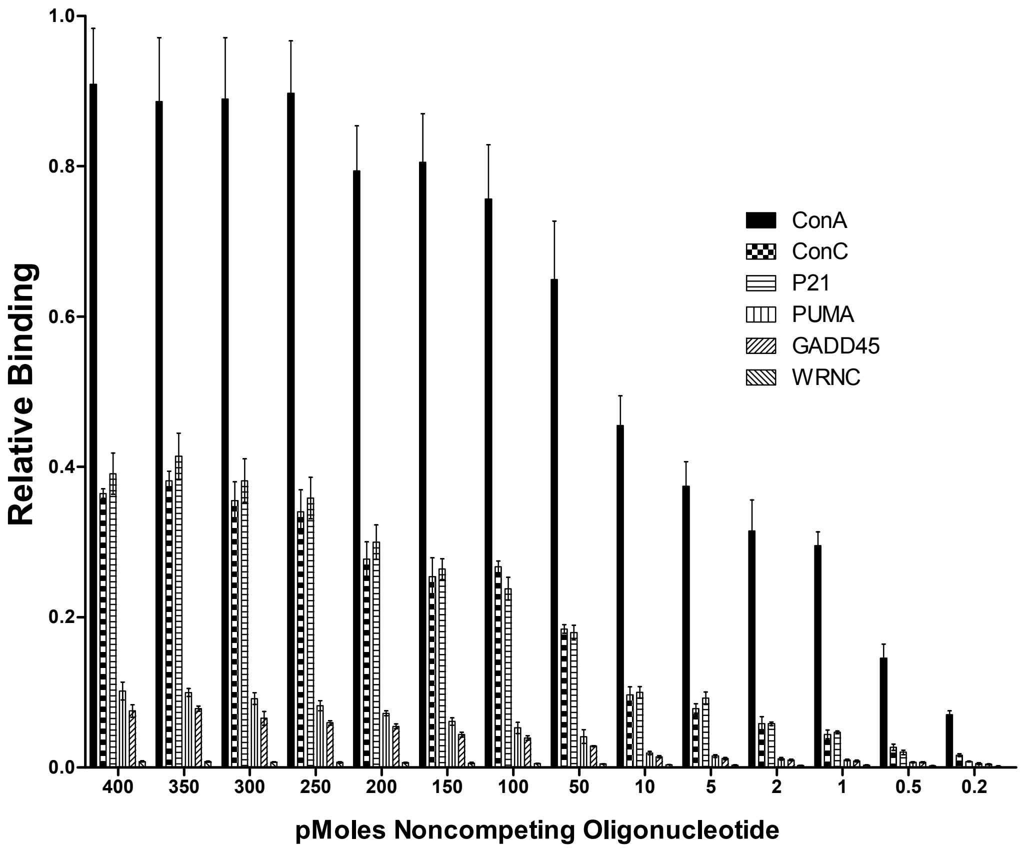

B Competing Oligonucleotide Abolishes Binding

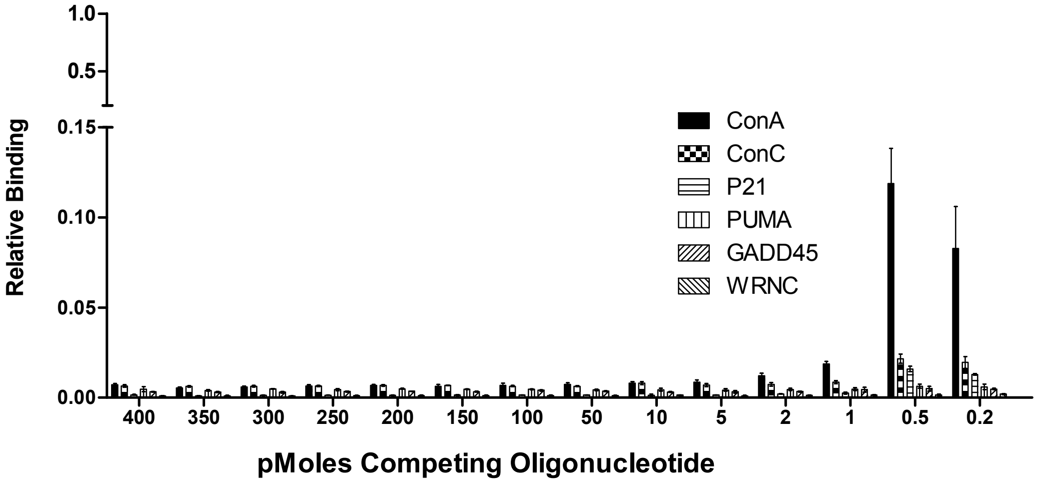

Supplement: Figure S3 — p53 binding in the presence of non-competing and competing oligonucleotides. Specificity was ascertained by varying amounts of competing oligonucleotide which was observed to reduce specific binding in a concentration-dependent manner. Similarly, the use of a noncompeting (or nonspecific) oligonucleotide, which blocks binding of nonspecific DNA binding proteins to the target sequences, enhances the sensitivity as well as detection limit and dynamic range of the assay. A multiplex set of 6 oligonucleotide-conjugated beads, 5 of which carry p53 REs (ConA, ConC, P21, PUMA, GADD45) and a negative control (WRNC), were incubated with nuclear extracts (NT: non-treated; Doxo: Doxorubicin-treated) for 60 minutes in the presence of variable amounts of either non-competing (NC) or competing (Comp) oligonucleotides and analyzed for p53 binding (see Materials and Methods for sequence of oligonucleotides used). A) The oligonucleotide set was incubated with decreasing amounts of non-competing oligonucleotide (shown in pmoles per reaction) and 1.75 micrograms of nuclear extracts from Doxo-treated cells. B) Treatment with decreasing amounts of a competing oligonucleotide and 1.75 micrograms of nuclear extract from Doxo-treated cells. NC oligonucleotides were not added in this experiment. Under these conditions ∼1 pmole of competing oligonucleotide is needed to eliminate p53 binding. The relative binding intensity (value shown on vertical axes) was obtained for each oligonucleotide as discussed in Materials and Methods. Note breaks in the scale of vertical axes. Values shown are means for each bead type±SD (n = 3). (0.06 MB PDF) [file pgen.1000462.s003.pdf]
