# Supplementary material for: Probing the Functional Impact of Sequence Variation on p53-DNA Interactions Using a Novel Microsphere Assay for Protein-DNA Binding with Human Cell Extracts
Source: PLoS Genet. 2009 May 8;5(5):e1000462. doi: 10.1371/journal.pgen.1000462 (PMC2667269; doi:10.1371/journal.pgen.1000462)
Supplement: Figure S4 — Concentration versus binding isotherm for nuclear extracts containing activated p53. A multiplex set of 6 oligonucleotide-conjugated beads, five carrying p53 REs (ConA, ConC, P21, PUMA, GADD45) and one a negative control (WRNC), was incubated with variable amounts of DOXO treated nuclear extracts in the presence of 150 pmoles of non-competing oligonucleotides for 60 minutes and analyzed for p53 binding. A) Normalized binding for each oligonucleotide is shown with varying amounts of nuclear extracts from Doxo-treated cells. The relative binding intensity (value shown on vertical axes) was obtained for each oligonucleotide as discussed in Materials and Methods. Values shown are means for each bead type±SD (n = 3). Nonlinear regression was used to determine BMax and Kd. A concentration of nuclear extract that was saturating for the p21 RE (1.75 ug) was chosen for the assay. An additional parameter investigated included the impact of bead quantity on relative binding. Bead quantity was varied from 2500 to 40,000 beads (representing up to 80 bead types), each bead represented at equal concentration. These experiments were carried out under varying protein concentrations. Using 1.75 ug protein in the hybridization, no effect of bead number on binding was observed. At lower protein concentrations (less than 1.2 ug protein) binding values were reduced for higher bead quantities in a linear, bead-quantity dependent manner. Thus protein and bead/probe number need to be optimized for a given system. (0.05 MB PDF) [file pgen.1000462.s004.pdf]

Doxo p53 Binding Isotherm

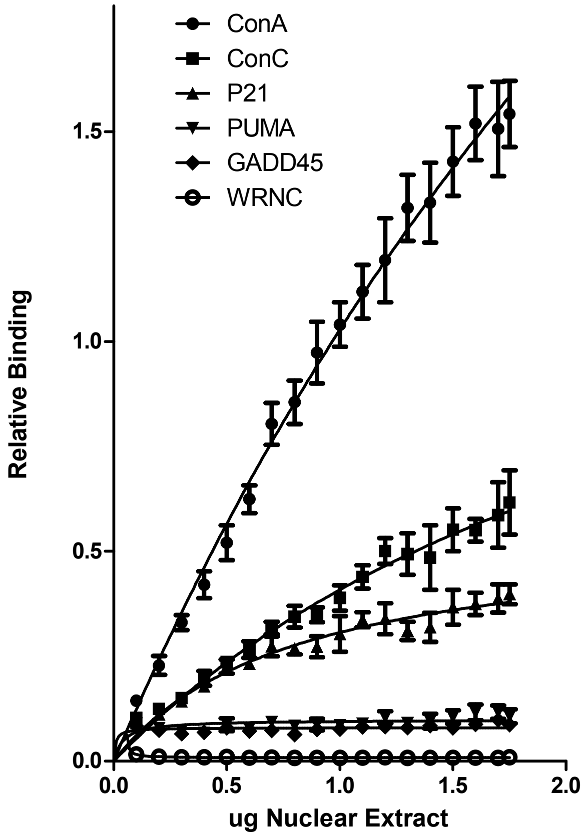

Best-fit values	ConA	ConC	P21	PUMA	GADD45	WRNC
BMAX	5.657	1.539	0.5515	0.0984	0.0792	0.00778
KD	4.502	2.769	0.7989	0.0435	0.00784	-0.0515
Std. Error						
BMAX	0.6276	0.1878	0.0320	0.0048	0.00279	0.00014
KD	0.6429	0.4906	0.1081	0.0256	0.01307	0.00212
Goodness of Fit						
Degrees of Freedom	16	16	16	16	16	16
R^2	0.995	0.984	0.967	0.175	0.0202	0.915
