# Supplementary material for: Probing the Functional Impact of Sequence Variation on p53-DNA Interactions Using a Novel Microsphere Assay for Protein-DNA Binding with Human Cell Extracts
Source: PLoS Genet. 2009 May 8;5(5):e1000462. doi: 10.1371/journal.pgen.1000462 (PMC2667269; doi:10.1371/journal.pgen.1000462)
Supplement: Table S1 — Oligonucleotide sequences. (0.07 MB DOC) [file pgen.1000462.s009.doc]

**Table S1. Oligonucleotide sequences for**

**Figure 2 Oligonucleotide set**

**Oligo Name Luminex Bead # Tag Sequence Oligonucleotide Sequence**

**ConA 57-** CAATATCATCATCTTTATCATTAC **CTTTCTGGCCATCATGGGCATGTCCGGGCATGTCCAGCTCTGGCATAGAA**

**CONC 74**- TACACATCTTACAAACTAATTTCA **CTTTCTGGCCATCATGGGCAAGTCTGGGCAAGTCTAGCTCTGGCATAGAA**

**P21 14**- CTACTATACATCTTACTATACTTT **CTTTCTGGCCATCATGAACATGTCCCAACATGTTGAGCTCTGGCATAGAA**

**PUMA 68**- TCATAATCTCAACAATCTTTCTTT **CTTTCTGGCCATCATCTGCAAGTCCTGACTTGTCCAGCTCTGGCATAGAA**

**GADD45 23**- TTCAATCATTCAAATCTCAACTTT **CTTTCTGGCCATCATGAACATGTCTAAGCATGCTGAGCTCTGGCATAGAA**

**WRNC 13-** CAATAAACTATACTTCTTCACTAA **CTTTCTGGCCATCATGAAAGGTGGATTTAGGTGGAAGCTCTGGCATAGAA**

**Figure 3A Oligonucleotide set**

**ConA** 57**-** CAATATCATCATCTTTATCATTAC **CTTTCTGGCCATCATGGGCATGTCCGGGCATGTCCAGCTCTGGCATAGAA**

**P21LWT** 14- CTACTATACATCTTACTATACTTT  **CTTTCTGGCCATCATGAACATGTCCCAACATGTTGAGCTCTGGCATAGAA**

**P21GL3** 31- TTCACTTTTCAATCAACTTTAATC  **TACAGATGCACATATGAACATGTCCCAACATGTTGCGAGGTGGACATCAC**

**4C>T**  5- CAATTCAAATCACAATAATCAATC  **CTTTCTGGCCATCATGAATATGTCCCAACATGTTGAGCTCTGGCATAGAA**

**4C>A**  6- TCAACAATCTTTTACAATCAAATC  **CTTTCTGGCCATCATGAAAATGTCCCAACATGTTGAGCTCTGGCATAGAA**

**4C>G**  10- ATCATACATACATACAAATCTACA  **CTTTCTGGCCATCATGAAGATGTCCCAACATGTTGAGCTCTGGCATAGAA**

**7G>C**  17- CTTTAATCCTTTATCACTTTATCA  **CTTTCTGGCCATCATGAACATCTCCCAACATGTTGAGCTCTGGCATAGAA**

**7G>A** 19- TCAATCAATTACTTACTCAAATAC  **CTTTCTGGCCATCATGAACATATCCCAACATGTTGAGCTCTGGCATAGAA**

**7G>T**  21- AATCCTTTCTTTAATCTCAAATCA  **CTTTCTGGCCATCATGAACATTTCCCAACATGTTGAGCTCTGGCATAGAA**

**14C>T**  23- TTCAATCATTCAAATCTCAACTTT  **CTTTCTGGCCATCATGAACATGTCCCAATATGTTGAGCTCTGGCATAGAA**

**14C>A**  25- CTTTTCAATTACTTCAAATCTTCA  **CTTTCTGGCCATCATGAACATGTCCCAAAATGTTGAGCTCTGGCATAGAA**

**14C>G** 28- CTACAAACAAACAAACATTATCAA  **CTTTCTGGCCATCATGAACATGTCCCAAGATGTTGAGCTCTGGCATAGAA**

**17G>C**  39- TACACAATCTTTTCATTACATCAT  **CTTTCTGGCCATCATGAACATGTCCCAACATCTTGAGCTCTGGCATAGAA**

**17G>A**  48- AAACAAACTTCACATCTCAATAAT  **CTTTCTGGCCATCATGAACATGTCCCAACATATTGAGCTCTGGCATAGAA**

**17G>T**  62- TCAATCATAATCTCATAATCCAAT  **CTTTCTGGCCATCATGAACATGTCCCAACATTTTGAGCTCTGGCATAGAA**

**7G>C;17G>T** 66- TAACATTACAACTATACTATCTAC  **CTTTCTGGCCATCATGAACATCTCCCAACATTTTGAGCTCTGGCATAGAA**

**11C>G**  72- TCATTTACCTTTAATCCAATAATC  **CTTTCTGGCCATCATGAACATGTCCGAACATGTTGAGCTCTGGCATAGAA**

**11C>G;20G>C** 74- TACACATCTTACAAACTAATTTCA  **CTTTCTGGCCATCATGAACATGTCCGAACATGTTCAGCTCTGGCATAGAA**

**WRNC** 13**-** CAATAAACTATACTTCTTCACTAA  **CTTTCTGGCCATCATGAAAGGTGGATTTAGGTGGAAGCTCTGGCATAGAA**

**Figure 3B Oligonucleotide set**

**Oligo Name Luminex Bead # Tag Sequence Oligonucleotide Sequence**

**ConA**  57**-** CAATATCATCATCTTTATCATTAC **CTTTCTGGCCATCATGGGCATGTCCGGGCATGTCCAGCTCTGGCATAGAA**

**G>C**  12- TACACTTTCTTTCTTTCTTTCTTT **CTTTCTGGCCATCATCGGCATGTCCGGGCATGTCCAGCTCTGGCATAGAA**

**G>A**  49- TCATCAATCTTTCAATTTACTTAC **CTTTCTGGCCATCATAGGCATGTCCGGGCATGTCCAGCTCTGGCATAGAA**

**G>T**  59- TCATCAATCAATCTTTTTCACTTT **CTTTCTGGCCATCATTGGCATGTCCGGGCATGTCCAGCTCTGGCATAGAA**

**G>C**  68- TCATAATCTCAACAATCTTTCTTT **CTTTCTGGCCATCATGCGCATGTCCGGGCATGTCCAGCTCTGGCATAGAA**

**G>A**  14- CTACTATACATCTTACTATACTTT **CTTTCTGGCCATCATGAGCATGTCCGGGCATGTCCAGCTCTGGCATAGAA**

**G>T**  33- TCAATTACTTCACTTTAATCCTTT **CTTTCTGGCCATCATGTGCATGTCCGGGCATGTCCAGCTCTGGCATAGAA**

**G>C**  02- CTTTATCAATACATACTACAATCA **CTTTCTGGCCATCATGGCCATGTCCGGGCATGTCCAGCTCTGGCATAGAA**

**G>A**  42- CTATCTTCATATTTCACTATAAAC **CTTTCTGGCCATCATGGACATGTCCGGGCATGTCCAGCTCTGGCATAGAA**

**G>T**  08- AATCCTTTTACATTCATTACTTAC **CTTTCTGGCCATCATGGTCATGTCCGGGCATGTCCAGCTCTGGCATAGAA**

**C>A**  80- CTAACTAACAATAATCTAACTAAC **CTTTCTGGCCATCATGGGAATGTCCGGGCATGTCCAGCTCTGGCATAGAA**

**C>T**  51- TCATTTCAATCAATCATCAACAAT **CTTTCTGGCCATCATGGGTATGTCCGGGCATGTCCAGCTCTGGCATAGAA**

**C>G**  48- AAACAAACTTCACATCTCAATAAT **CTTTCTGGCCATCATGGGGATGTCCGGGCATGTCCAGCTCTGGCATAGAA**

**A>C**  65- CTTTTCATCAATAATCTTACCTTT **CTTTCTGGCCATCATGGGCCTGTCCGGGCATGTCCAGCTCTGGCATAGAA**

**A>G**  29- AATCTTACTACAAATCCTTTCTTT **CTTTCTGGCCATCATGGGCGTGTCCGGGCATGTCCAGCTCTGGCATAGAA**

**A>T**  82- TACATACACTAATAACATACTCAT **CTTTCTGGCCATCATGGGCTTGTCCGGGCATGTCCAGCTCTGGCATAGAA**

**T>C**  03- TACACTTTATCAAATCTTACAATC **CTTTCTGGCCATCATGGGCACGTCCGGGCATGTCCAGCTCTGGCATAGAA**

**T>G**  21- AATCCTTTCTTTAATCTCAAATCA **CTTTCTGGCCATCATGGGCAGGTCCGGGCATGTCCAGCTCTGGCATAGAA**

**T>A**  11- TACAAATCATCAATCACTTTAATC **CTTTCTGGCCATCATGGGCAAGTCCGGGCATGTCCAGCTCTGGCATAGAA**

**G>C**  22- AATCCTTTTTACTCAATTCAATCA **CTTTCTGGCCATCATGGGCATCTCCGGGCATGTCCAGCTCTGGCATAGAA**

**G>T**  100- CTATCTTTAAACTACAAATCTAAC **CTTTCTGGCCATCATGGGCATTTCCGGGCATGTCCAGCTCTGGCATAGAA**

**G>A**  81- CTTTAATCTACACTTTCTAACAAT **CTTTCTGGCCATCATGGGCATATCCGGGCATGTCCAGCTCTGGCATAGAA**

**T>A**  37- CTTTTCATCTTTTCATCTTTCAAT **CTTTCTGGCCATCATGGGCATGACCGGGCATGTCCAGCTCTGGCATAGAA**

**T>C**  20- CTTTTACAATACTTCAATACAATC **CTTTCTGGCCATCATGGGCATGCCCGGGCATGTCCAGCTCTGGCATAGAA**

**T>G**  39- TACACAATCTTTTCATTACATCAT **CTTTCTGGCCATCATGGGCATGGCCGGGCATGTCCAGCTCTGGCATAGAA**

**C>T**  23- TTCAATCATTCAAATCTCAACTTT **CTTTCTGGCCATCATGGGCATGTTCGGGCATGTCCAGCTCTGGCATAGAA**

**C>A**  31- TTCACTTTTCAATCAACTTTAATC **CTTTCTGGCCATCATGGGCATGTACGGGCATGTCCAGCTCTGGCATAGAA**

**C>G**  01- CTTTAATCTCAATCAATACAAATC **CTTTCTGGCCATCATGGGCATGTGCGGGCATGTCCAGCTCTGGCATAGAA**

**C>T**  89- TATACTATCAACTCAACAACATAT **CTTTCTGGCCATCATGGGCATGTCTGGGCATGTCCAGCTCTGGCATAGAA**

**C>A**  05- CAATTCAAATCACAATAATCAATC **CTTTCTGGCCATCATGGGCATGTCAGGGCATGTCCAGCTCTGGCATAGAA**

**C>G**  67- TCATTTACTCAACAATTACAAATC **CTTTCTGGCCATCATGGGCATGTCGGGGCATGTCCAGCTCTGGCATAGAA**

**G>C**  71- ATCATTACAATCCAATCAATTCAT **CTTTCTGGCCATCATGGGCATGTCCCGGCATGTCCAGCTCTGGCATAGAA**

**Figure 3B Oligonucleotide set cont.**

**Oligo Name Luminex Bead # Tag Sequence Oligonucleotide Sequence**

**G>T**  58- CTACTAATTCATTAACATTACTAC **CTTTCTGGCCATCATGGGCATGTCCTGGCATGTCCAGCTCTGGCATAGAA**

**G>A**  07- CAATTCATTTACCAATTTACCAAT **CTTTCTGGCCATCATGGGCATGTCCAGGCATGTCCAGCTCTGGCATAGAA**

**G>C**  24- TCAATTACCTTTTCAATACAATAC **CTTTCTGGCCATCATGGGCATGTCCGCGCATGTCCAGCTCTGGCATAGAA**

**G>T**  84- TCAACTAACTAATCATCTATCAAT **CTTTCTGGCCATCATGGGCATGTCCGTGCATGTCCAGCTCTGGCATAGAA**

**G>A**  28- CTACAAACAAACAAACATTATCAA **CTTTCTGGCCATCATGGGCATGTCCGAGCATGTCCAGCTCTGGCATAGAA**

**G>C**  32- ATTATTCACTTCAAACTAATCTAC **CTTTCTGGCCATCATGGGCATGTCCGGCCATGTCCAGCTCTGGCATAGAA**

**G>T**  25- CTTTTCAATTACTTCAAATCTTCA **CTTTCTGGCCATCATGGGCATGTCCGGTCATGTCCAGCTCTGGCATAGAA**

**G>A**  55- TATATACACTTCTCAATAACTAAC **CTTTCTGGCCATCATGGGCATGTCCGGACATGTCCAGCTCTGGCATAGAA**

**C>A**  56- CAATTTACTCATATACATCACTTT **CTTTCTGGCCATCATGGGCATGTCCGGGAATGTCCAGCTCTGGCATAGAA**

**C>T**  34- TCATTCATATACATACCAATTCAT **CTTTCTGGCCATCATGGGCATGTCCGGGTATGTCCAGCTCTGGCATAGAA**

**C>G**  26- TTACTCAAAATCTACACTTTTTCA **CTTTCTGGCCATCATGGGCATGTCCGGGGATGTCCAGCTCTGGCATAGAA**

**A>C**  91- TTCATAACATCAATCATAACTTAC **CTTTCTGGCCATCATGGGCATGTCCGGGCCTGTCCAGCTCTGGCATAGAA**

**A>G**  66- TAACATTACAACTATACTATCTAC **CTTTCTGGCCATCATGGGCATGTCCGGGCGTGTCCAGCTCTGGCATAGAA**

**A>T**  30- TTACCTTTATACCTTTCTTTTTAC **CTTTCTGGCCATCATGGGCATGTCCGGGCTTGTCCAGCTCTGGCATAGAA**

**T>C**  87- AAACTAACATCAATACTTACATCA **CTTTCTGGCCATCATGGGCATGTCCGGGCACGTCCAGCTCTGGCATAGAA**

**T>G**  72- TCATTTACCTTTAATCCAATAATC **CTTTCTGGCCATCATGGGCATGTCCGGGCAGGTCCAGCTCTGGCATAGAA**

**T>A**  19- TCAATCAATTACTTACTCAAATAC **CTTTCTGGCCATCATGGGCATGTCCGGGCAAGTCCAGCTCTGGCATAGAA**

**G>C**  41- TTACTACACAATATACTCATCAAT **CTTTCTGGCCATCATGGGCATGTCCGGGCATCTCCAGCTCTGGCATAGAA**

**G>T**  79- TTCATAACTACAATACATCATCAT **CTTTCTGGCCATCATGGGCATGTCCGGGCATTTCCAGCTCTGGCATAGAA**

**G>A**  27- CTTTTCAAATCAATACTCAACTTT **CTTTCTGGCCATCATGGGCATGTCCGGGCATATCCAGCTCTGGCATAGAA**

**T>A**  10- ATCATACATACATACAAATCTACA **CTTTCTGGCCATCATGGGCATGTCCGGGCATGACCAGCTCTGGCATAGAA**

**T>C**  45- TCATTTCACAATTCAATTACTCAA **CTTTCTGGCCATCATGGGCATGTCCGGGCATGCCCAGCTCTGGCATAGAA**

**T>G**  06- TCAACAATCTTTTACAATCAAATC **CTTTCTGGCCATCATGGGCATGTCCGGGCATGGCCAGCTCTGGCATAGAA**

**C>T**  36- CAATTCATTTCATTCACAATCAAT **CTTTCTGGCCATCATGGGCATGTCCGGGCATGTTCAGCTCTGGCATAGAA**

**C>A**  35- CAATTTCATCATTCATTCATTTCA **CTTTCTGGCCATCATGGGCATGTCCGGGCATGTACAGCTCTGGCATAGAA**

**C>G**  86- CTAATTACTAACATCACTAACAAT **CTTTCTGGCCATCATGGGCATGTCCGGGCATGTGCAGCTCTGGCATAGAA**

**C>T**  15- ATACTTCATTCATTCATCAATTCA **CTTTCTGGCCATCATGGGCATGTCCGGGCATGTCTAGCTCTGGCATAGAA**

**C>A**  62- TCAATCATAATCTCATAATCCAAT **CTTTCTGGCCATCATGGGCATGTCCGGGCATGTCAAGCTCTGGCATAGAA**

**C>G**  74- TACACATCTTACAAACTAATTTCA **CTTTCTGGCCATCATGGGCATGTCCGGGCATGTCGAGCTCTGGCATAGAA**

**WRNC**  13- CAATAAACTATACTTCTTCACTAA **CTTTCTGGCCATCATGAAAGGTGGATTTAGGTGGAAGCTCTGGCATAGAA**

**Figures 4C, 4D and 6A, 6B Oligonucleotide set (names as in Inga et al.[4])**

**Oligo Name (Gene) Luminex Bead # Tag Sequence Oligonucleotide Sequence**

**ConA**  57**-** CAATATCATCATCTTTATCATTAC **CTTTCTGGCCATCATGGGCATGTCCGGGCATGTCCAGCTCTGGCATAGAA**

**P21-5 (CDKN1A)** 01-CTTTAATCTCAATCAATACAAATC **CTTTCTGGCCATCATCAACATGTTGGGACATGTTCAGCTCTGGCATAGAA**

**P21LT (CDKN1A)** 14-CTACTATACATCTTACTATACTTT **CTTTCTGGCCATCATGAACATGTCCCAACATGTTGAGCTCTGGCATAGAA**

**P53R2 (RRM2B)** 21-AATCCTTTCTTTAATCTCAAATCA **CTTTCTGGCCATCATTGACATGCCCAGGCATGTCTAGCTCTGGCATAGAA**

**m-Fas (mFas)** 05-CAATTCAAATCACAATAATCAATC **CTTTCTGGCCATCATGGGCATGTACAAACATGTCAAGCTCTGGCATAGAA**

**GADD45 (GADD45)** 23-TTCAATCATTCAAATCTCAACTTT **CTTTCTGGCCATCATGAACATGTCTAAGCATGCTGAGCTCTGGCATAGAA**

**ConB**  66-TAACATTACAACTATACTATCTAC **CTTTCTGGCCATCATGGGCTAGTCCGGGCTAGTCCAGCTCTGGCATAGAA**

**HFAS (FAS)** 25-CTTTTCAATTACTTCAAATCTTCA **CTTTCTGGCCATCATTGGCTTGTCAGGGCTTGTCCAGCTCTGGCATAGAA**

**ConC**  65-CTTTTCATCAATAATCTTACCTTT **CTTTCTGGCCATCATGGGCAAGTCTGGGCAAGTCTAGCTCTGGCATAGAA**

**1433σ1 (SFN)** 39-TACACAATCTTTTCATTACATCAT **CTTTCTGGCCATCATTAGCATGCCCAGACATGTCCAGCTCTGGCATAGAA**

**PCNA (PCNA)** 06-TCAACAATCTTTTACAATCAAATC **CTTTCTGGCCATCATGAACAAGTCCGGGCATATGTAGCTCTGGCATAGAA**

**AIP1 (P53AIP1)**  19-TCAATCAATTACTTACTCAAATAC **CTTTCTGGCCATCATTCTCTTGCCCGGGCTTGTCGAGCTCTGGCATAGAA**

**PUMA (BBC3)** 49-TCATCAATCTTTCAATTTACTTAC **CTTTCTGGCCATCATCTGCAAGTCCTGACTTGTCCAGCTCTGGCATAGAA**

**MutRGC**  28-CTACAAACAAACAAACATTATCAA **CTTTCTGGCCATCATGGACATGCCTGGCCATGCCTAGCTCTGGCATAGAA**

**CycG (Cnng1)** 37-CTTTTCATCTTTTCATCTTTCAAT **CTTTCTGGCCATCATAGGCTTGCCCGGGCAGGTCTAGCTCTGGCATAGAA**

**NOXA (PMAIP1)** 56-CAATTTACTCATATACATCACTTT **CTTTCTGGCCATCATAGGCTTGCCCCGGCAAGTTGAGCTCTGGCATAGAA**

**P21-3’ (CDKN1A)** 30-TTACCTTTATACCTTTCTTTTTAC **CTTTCTGGCCATCATGAAGAAGACTGGGCATGTCTAGCTCTGGCATAGAA**

**MDM2RE1 (MDM2)** 26-TTACTCAAAATCTACACTTTTTCA **CTTTCTGGCCATCATGGTCAAGTTGGGACACGTCCAGCTCTGGCATAGAA**

**PA26 (SESN1)** 35-CAATTTCATCATTCATTCATTTCA **CTTTCTGGCCATCATGGACAAGTCTCAACAAGTTCAGCTCTGGCATAGAA**

**BAX-B (BAX)** 42-CTATCTTCATATTTCACTATAAAC **CTTTCTGGCCATCATAGACAAGCCTGGGCGTGGGCAGCTCTGGCATAGAA**

**MDM2RE2 (MDM2)** 72-TCATTTACCTTTAATCCAATAATC **TTTCTGGCCATCATGAGCTAAGTCCTGACATGTCTAGCTCTGGCATAGAA**

**cFOS (mFos)** 17-CTTTAATCCTTTATCACTTTATCA **CTTTCTGGCCATCATGGACTTGTCTGAGCGCGTGCAGCTCTGGCATAGAA**

**RGC (RPLZ7A)** 03-TACACTTTATCAAATCTTACAATC **CTTTCTGGCCATCATGGACTTGCCTGGCCTTGCCTAGCTCTGGCATAGAA**

**IGFBP3 (IGFBP3B)** 62-TCAATCATAATCTCATAATCCAAT **TTTCTGGCCATCATAAACAAGCCACCAACATGCTTAGCTCTGGCATAGAA**

**PIG3 (TP53I3)** 10-ATCATACATACATACAAATCTACA **CTTTCTGGCCATCATCAGCTTGCCCACCCATGCTCAGCTCTGGCATAGAA**

**BAX-A (BAX)**  43-CTTTCAATTACAATACTCATTACA **TTTCTGGCCATCATTCACAAGTTAGAGACAAGCCTAGCTCTGGCATAGAA**

**IGFBP3B (IGFBP3B)** 11-TACAAATCATCAATCACTTTAATC **CTTTCTGGCCATCATGGGCAAGACCTGCCAAGCCTAGCTCTGGCATAGAA**

**WRNC**  13**-** CAATAAACTATACTTCTTCACTAA **CTTTCTGGCCATCATGAAAGGTGGATTTAGGTGGAAGCTCTGGCATAGAA**

***Note that for REs containing spacers >0 (MDM2RE2, IFGBP3 and BAX-A), the nonbinding flanking sequence was shortened.**

**Figure 5 and Figure S8 Oligonucleotide set**

**Oligo Name Luminex Bead # Tag Sequence Oligonucleotide Sequence**

WRNC 15- TACACTTTCTTTCTTTCTTTCTTT  **CTTTCTGGCCATCATGAAAGGTGGATTTAGGTGGAAGCTCTGGCATAGAA**

P21wt 20- CTTTTACAATACTTCAATACAATC **CTTTCTGGCCATCATGAACATGTCCCAACATGTTGAGCTCTGGCATAGAA**

P21mt 73- CTTTTACAATACTTCAATACAATC **CTTTCTGGCCATCATGAACATTTCCCAACATGTTGAGCTCTGGCATAGAA**

ADARB1 Strong 12- TACACTTTCTTTCTTTCTTTCTTT **CTTTCTGGCCATCATGGACAAGTTGAAACTTGCACAGCTCTGGCATAGAA**

ADARB1 Weak 12- TACACTTTCTTTCTTTCTTTCTTT **CTTTCTGGCCATCATGGACAAGTTGAAACTTACACAGCTCTGGCATAGAA**

ARHGEF7 Strong 25- CTTTTCAATTACTTCAAATCTTCA **CTTTCTGGCCATCATAAACATGTCAGCACTTGCTTAGCTCTGGCATAGAA**

ARHGEF7 Weak 25- CTTTTCAATTACTTCAAATCTTCA **CTTTCTGGCCATCATAAACATGTCATCACTTGCTTAGCTCTGGCATAGAA**

DCC Strong 26- TTACTCAAAATCTACACTTTTTCA **CTTTCTGGCCATCATGAGCATGTTCACACAAGCCAAGCTCTGGCATAGAA**

DCC Weak 26- TTACTCAAAATCTACACTTTTTCA **CTTTCTGGCCATCATCAGCATGTTCACACAAGCCAAGCTCTGGCATAGAA**

EOMES Strong 39- TACACAATCTTTTCATTACATCAT **CTTTCTGGCCATCATGGGCCTGTCTCAACTTGCCCAGCTCTGGCATAGAA**

EOMES Weak 39- TACACAATCTTTTCATTACATCAT **CTTTCTGGCCATCATGGGCCTGTCTCAACTCGCCCAGCTCTGGCATAGAA**

RRM1 Strong 65- CTTTTCATCAATAATCTTACCTTT **CTTTCTGGCCATCATGGGCATGTGCATTCAAGTTTAGCTCTGGCATAGAA**

RRM1 Weak 65- CTTTTCATCAATAATCTTACCTTT **CTTTCTGGCCATCATGGGTATGTGCATTCAAGTTTAGCTCTGGCATAGAA**

SCGBID1 Strong 66- TAACATTACAACTATACTATCTAC **CTTTCTGGCCATCATGGTCTTGTTTAGACTTGCTCAGCTCTGGCATAGAA**
SCGBID1 Weak 66- TAACATTACAACTATACTATCTAC **CTTTCTGGCCATCATGGTCTTGTTTAGACTTACTCAGCTCTGGCATAGAA**

SEI1 Strong 89- TATACTATCAACTCAACAACATAT **CTTTCTGGCCATCATGGGCTTGAGGGCGCATGCCCAGCTCTGGCATAGAA**

SEI1 Weak 89- TATACTATCAACTCAACAACATAT **CTTTCTGGCCATCATGGGCTTCAGGGCGCATGCCCAGCTCTGGCATAGAA**

TLR8 Strong 100- CTATCTTTAAACTACAAATCTAAC **CTTTCTGGCCATCATAGGCAAGATGAAACATGTCAAGCTCTGGCATAGAA**

TLR8 Weak 100- CTATCTTTAAACTACAAATCTAAC **CTTTCTGGCCATCATAGGCAAGATGAAACATATCAAGCTCTGGCATAGAA**

* “Strong” and “Weak” refer to predicted binding based on position weight matrix analysis.

**Figure S5 Oligonucleotide set**

**Oligo Name Luminex Bead # Tag Sequence Oligonucleotide Sequence**

**EBAG9**  31- TTCACTTTTCAATCAACTTTAATC **CTTTCTGGCCATCATGCGGGTCAGGGTGACCTCGAAGCTCTGGCATAGAA**

**EFP**  43- CTTTCAATTACAATACTCATTACA **CTTTCTGGCCATCATGAGGGTCATGGTGACCCTGAAGCTCTGGCATAGAA**

**COX7RP**  48- AAACAAACTTCACATCTCAATAAT **CTTTCTGGCCATCATGGGGGTCAAGGTGACCCCGAAGCTCTGGCATAGAA**

**TERT**  51- TCATTTCAATCAATCATCAACAAT **CTTTCTGGCCATCATGTTGGTCAGGCTGATCTCGAAGCTCTGGCATAGAA**

**pS2**  74- TACACATCTTACAAACTAATTTCA **CTTTCTGGCCATCATGAAGGTCACGGTGGCCACGAAGCTCTGGCATAGAA**

**LTF**  72- TCATTTACCTTTAATCCAATAATC **CTTTCTGGCCATCATGCAGGTCAAGGCGATCTTGAAGCTCTGGCATAGAA**

**KRT19**  81- CTTTAATCTACACTTTCTAACAAT **CTTTCTGGCCATCATGTAGGTCAGTAAGACCTCGAAGCTCTGGCATAGAA**

**ACE**  100- CTATCTTTAAACTACAAATCTAAC **CTTTCTGGCCATCATGCGGGTCACGATGCCCTAGAAGCTCTGGCATAGAA**

**CTSD**  20- CTTTTACAATACTTCAATACAATC **CTTTCTGGCCATCATGCCGGTCACGTGGGCGCGGAAGCTCTGGCATAGAA**

**ConA**  57- CAATATCATCATCTTTATCATTAC **CTTTCTGGCCATCATGGGCATGTCCGGGCATGTCCAGCTCTGGCATAGAA**

**WRNC**  12- TACACTTTCTTTCTTTCTTTCTTT **CTTTCTGGCCATCATGAAAGGTGGATTTAGGTGGAAGCTCTGGCATAGAA**
