# Supplementary material for: Probing the Functional Impact of Sequence Variation on p53-DNA Interactions Using a Novel Microsphere Assay for Protein-DNA Binding with Human Cell Extracts
Source: PLoS Genet. 2009 May 8;5(5):e1000462. doi: 10.1371/journal.pgen.1000462 (PMC2667269; doi:10.1371/journal.pgen.1000462)
Supplement: Table S2 — p53 response elements used to build position weight matrix. (0.10 MB DOC) [file pgen.1000462.s010.doc]

Table S2: Experimentally discovered p53 response elements for building PWM

| RE_name | Sequence (5’-half 3’half) | Chromosomal position | PWM score | gene |
| --- | --- | --- | --- | --- |
| ADARB1 | GTGCAAGTTT CAACTTGTCC | chr21:45316682-45316701 | 8.1 | ADARB1 |
| APAF1 | AGACATGTCT CGACAAGCCC | chr12:97540942-97540974 | 13.5 | APAF1 |
| ARHGEF7 | AAACATGTCA GCACTTGCTT | chr13:110602821-110602840 | 7.1 | ARHGEF7 |
| ATF3 | AGTCATGCCG GGTCATGCCT | chr1:209170119-209170155 | 11 | ATF3 |
| BAX_B | AGACAAGCCT GGGCGTGGGC | chr19:54149524-54149543 | 7.1 | BAX |
| BBC3 | CTGCAAGTCC TGACTTGTCC | chr19:52426417-52426436 | 9.3 | BBC3 |
| BID | GGGCATGATG GTGCATGCCT | chr22:16614037-16614056 | 9.9 | BID |
| C12ORF5 | AGACAAGTCT GGACATGTCT | chr12:4301042-4301063 | 13.5 | C12ORF5 |
| C13orf15 | AGGCGAGTTT CAGCTTGTCC | chr13:40930827-40930849 | 7.3 | C13orf15 |
| CASP1 | AGACATGCAT ATGCATGCAC | chr11:104411147-104411166 | 7.6 | CASP1 |
| CASP6 | AGGCAAGGAG AGACAAGTCT | chr4:110976236-110976259 | 7.3 | CASP6 |
| CDKN1A | GAACATGTCC CAACATGTTG | chr6:36752204-36752223 | 10 | CDKN1A |
| CDKN1A | AAACATGCCC CAACAAGCTG | chr6:36750474-36750504 | 9.7 | CDKN1A |
| CDKN1A | GAAGAAGACT GGGCATGTCT | chr6:36753086-36753105 | 7.8 | CDKN1A |
| CX3CL1 | GGGCATGTTC CAGCTTGTGG | chr16:55963635-55963655 | 7.8 | CX3CL1 |
| DCC1 | CAGCATGTTC ACACAAGCCA | chr18:48118859-48118878 | 7.3 | DCC1 |
| DDB2 | AAGCTGGTTT GAACAAGCCC | chr11:47193096-47193115 | 8.2 | DDB2 |
| DSC3 | GAAGTTGCTC AGGCAAGCCT | chr18:26876754-26876779 | 7.9 | DSC3 |
| EDN2 | CTGCAAGCCC GGGCATGCCC | chr1:41617174-41617193 | 13.7 | EDN2 |
| EOMES | GGGCCTGTCT CAACTTGCCC | chr3:27739623-27739643 | 10.9 | EOMES |
| FAS | GGACAAGCCC TGACAAGCCA | chr10:90741046-90741065 | 11.5 | FAS |
| FDXR | GGGCAGGAGC GGGCTTGCCC | chr17:70380716-70380735 | 9.4 | FDXR |
| GADD45A | GAACATGTCT AAGCATGCTG | chr1:67864479-67864498 | 10.6 | GADD45A |
| GDF15 | AGCCATGCCC GGGCAAGAAC | chr19:18357996-18358015 | 9.1 | GDF15 |
| GDF15 | CATCTTGCCC AGACTTGTCT | chr19:18357118-18357137 | 9.2 | GDF15 |
| GPX1 | GGGCCAGACC AGACATGCCT | chr3:49370958-49370977 | 11.1 | GPX1 |
| IBRDC2 | AGACAGGTCC TGACAAGCAG | chr6:18495404-18495423 | 7.3 | IBRDC2 |
| IGFBP3 | GGGCAAGACC TGCCAAGCCT | chr7:45730002-45730021 | 9.2 | IGFBP3 |
| IGFBP3 | AAACAAGCCA CAACATGCTT | chr7:45730921-45730941 | 7.9 | IGFBP3 |
| LRDD | AGGCCTGCCT GGACATGTCT | chr11:794414-794441 | 12.8 | LRDD |
| MMP2 | AGACAAGCCT GAACTTGTCT | chr16:54068958-54068977 | 11.9 | MMP2 |
| P53AIP1 | TCTCTTGCCC GGGCTTGTCG | chr11:128316011-128316030 | 7.8 | P53AIP1 |
| PDGFC | GGTCATGTTC AGACTTGCCC | chr4:158050367-158050386 | 11.6 | PDGFC |
| PERP | AGGCAAGCTC CAGCTTGTTC | chr6:138470600-138470625 | 9.6 | PERP |
| PLK2 | AAACATGCCT GGACTTGCCC | chr5:57793858-57793877 | 13.6 | PLK2 |
| PMAIP1 | GAGCGTGTCC GGGCAGGTCG | chr18:55718023-55718042 | 9.7 | PMAIP1 |
| PRKAB1 | GTTCTTGCCG CGGCTTGCCT | chr12:118568545-118568564 | 8.4 | PRKAB1 |
| PTEN | GAGCAAGCCC GGGCATGCTC | chr10:89613057-89613090 | 14.4 | PTEN |
| RPS27L | GGGCATGTAG TGACTTGCCC | chr15:61236487-61236506 | 10.1 | RPS27L |
| RRM2B | TGACATGCCC AGGCATGTCT | chr8:103318244-103318263 | 13.5 | RRM2B |
| SCARA3 | GGGCAAGCCC AGACAAGTTG | chr8:27564569-27564588 | 12.4 | SCARA3 |
| SCGB1D2 | GGTCTTGTTT AGACTTGCTC | chr11:61765841-61765860 | 8.5 | SCGB1D2 |
| SEMA3B | TTGCATGCCC AGACATGTCT | chr3:50280796-50280817 | 11.2 | SEMA3B |
| SERPINE1 | ACACATGCCT CAGCAAGTCC | chr7:100363660-100363679 | 10.4 | SERPINE1 |
| SESN1 | GGACAAGTCT CCACAAGTCT | chr6:109436914-109436933 | 10.2 | SESN1 |
| SFN_BDS2 | GCATTAGCCC AGACATGTCC | chr1:27060408-27060427 | 7.4 | SFN |
| SIVA | GTACTTGGCA GGGCATGTCT | chr14:103985698-103985717 | 8.2 | SIVA |
| SOD2 | GTGCTTGTTC GGGCATGTCC | chr6:160086755-160086779 | 12 | SOD2 |
| TGFA | GGGCAGGCCC TGCCTAGTCT | chr2:70692650-70692669 | 8.5 | TGFA |
| TNFRSF10B | GGGCATGTCC GGGCAAGACG | chr8:22982080-22982099 | 13.3 | TNFRSF10B |
| TNFRSF10C | GGGCATGTCC GGGCAGGACG | chr8:23016747-23016766 | 11.9 | TNFRSF10C |
| TP53I3 | CAGCTTGCCC ACCCATGCTC | chr2:24219686-24219705 | 7.6 | TP53I3 |
| TP73 | GGGCAAGCTG AGGCCTGCCC | chr1:3630317-3630336 | 11.6 | TP73 |
| TP73 | GGGCAAGCTG GGACTTGGAT | chr1:3597020-3597050 | 7.4 | TP73 |
| TRAF4 | GGGCAAGCCA GGGCCTGCCT | chr17:24094291-24094310 | 12.1 | TRAF4 |
| UBTD1 | GAGCAAGCCC AGACTTGTCA | chr10:99299918-99299937 | 11.2 | UBTD1 |
| UNC5B_BS_A | GGACCTGTCT GATCACGCCC | chr10:72676747-72676766 | 7.5 | UNC5B |
| XRCC5 | GAACTAGTTT AAACATGTTC | chr2:216781166-216781186 | 7.9 | XRCC5 |
| WIG1 | AAACAAGTCC AGACATGCCT | chr3:180270678-180270697 | 12.8 | ZMAT3 |
